# Galvanostatic Electroshock Synthesis of Low Loading Au−Pt Nanoalloys Onto Gas Diffusion Electrodes as Multifunctional Electrocatalysts for a Glycerol‐Fed Electrolyzer

**DOI:** 10.1002/cssc.202400996

**Published:** 2024-09-04

**Authors:** Zahra Hagheh Kavousi, Layal Abdallah, Massomeh Ghorbanloo, Valerie Bonniol, Bertrand Rebiere, David Cornu, Mikhael Bechelany, Yaovi Holade

**Affiliations:** ^1^ Institut Européen des Membranes, IEM, UMR 5635 Univ Montpellier, ENSCM, CNRS Montpellier France; ^2^ Department of Chemistry, Faculty of Sciences University of Zanjan P.O. Box Zanjan 4537138791 Iran; ^3^ Institut Charles Gerhardt, ICGM, UMR 5253 Univ Montpellier, ENSCM, CNRS Montpellier France; ^4^ French Research Network on Hydrogen (FRH2) Research Federation No. 2044 CNRS CNRS BP 32229 Nantes CEDEX 3 44322 France; ^5^ Functional Materials Group Gulf University for Science and Technology (GUST) Mubarak Al-Abdullah 32093 Kuwait

**Keywords:** anion exchange membrane, biomass, electrodeposition, gas diffusion electrode, glycerol

## Abstract

Water electrolysis is increasingly considered a viable solution for meeting the world′s growing energy demands and mitigating environmental issues. An inventive strategy to mitigate the energy requirements involves substituting the energy‐intensive oxygen evolution reaction (OER) with biomass‐derived glycerol electrooxidation. Nonetheless, the synthesis of electrocatalysts for controlling the selectivity towards added‐value chemicals at the anode and efficient H_2_ generation at the cathode remains a critical bottleneck. Herein, we implemented a galvanostatic electroshock synthesis approach to control the reduction kinetics of Au(III) and Pt(IV) to grow ultra‐low amount of gold‐platinum alloys on a gas diffusion electrode (12–26 μg_metal_ cm^−2^) for glycerol‐fed hydroxide anion exchange membrane based electrolyzer. The symmetric GDE‐Au_100–x_Pt_x_||GDE‐Au_100–x_Pt_x_ systems showed a notable improvement in electrolyzer performance (GDE‐Au_64_Pt_36_=201 mA cm^−2^) as compared to monometallic versions (GDE‐Au_100_Pt_0_=18 mA cm^−2^, GDE‐Au_0_Pt_100_=81 mA cm^−2^). Chromatography (HPLC) analysis underscores the critical importance of bulk electrolysis methodology (galvanostatic vs potentiostatic) for the efficient conversion of glycerol into high‐value‐added products. Regarding the electrical energy required to produce 1 kg of H_2_ for such an electrolyzer fed at the anode with glycerol, our results confirm a drastic decrease by a factor of at least two compared with conventional water electrolysis.

## Graphical abstract

We propose free‐standing electrocatalysts for glycerol oxidation as an alternative to the oxygen evolution at the anode. Using an electroshock synthesis, we achieve superior efficiency in glycerol‐fed electrolyzers, reducing energy needed for H_2_.

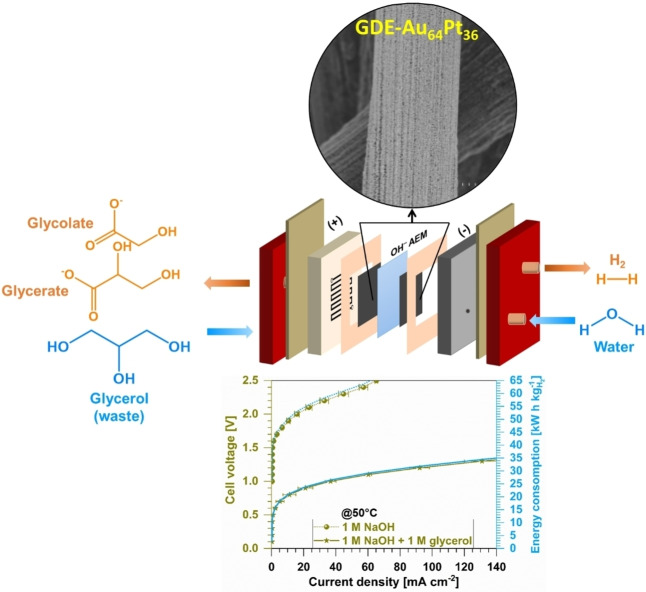

## Introduction

Addressing the urgent need for sustainable energy solutions requires innovative approaches that can mitigate dependence on fossil fuels. While hydrogen is often cited as a sustainable alternative, fossil fuels still account for more than 90 % of its production.^[1]^ Alkaline water electrolysis emerges as a viable candidate that not only meets growing energy demands but also minimizes environmental impact.^[4]^ In the presence of an externally applied voltage, water splits into hydrogen and oxygen, namely, hydrogen evolution reaction (HER) and oxygen evolution reaction (OER), at the anode and cathode, respectively.^[5]^ A major hurdle in current water electrolysis systems lies in their efficiency and cost‐effectiveness, particularly concerning OER part.[^1^, ^6^] Indeed, OER accounts for approximately 90 % of the energy consumed in hydrogen production via electrolysis, a consequence of its slow kinetics.^[8]^ This results in a high electricity consumption rate, expressed as *W*[kW h kg^−1^(H_2_)]=26.59×*U*[V].^[9]^ Additionally, the potential mixing of O_2_ with H_2_ in electrolytic systems can reduce production rates and safety issues, resulting in decreased energy efficiency and compromised operational reliability.^[10]^ In response to these challenges, the development of thermodynamically more favorable electrochemical oxidation of organic molecules, has garnered increasing research attention as substitutes for the OER.[^9^, ^13^]

In general, substrate molecules such as hydrazine, urea, glucose, and glycerol require lower oxidation potentials (*E*<1 V vs. RHE (reversible hydrogen electrode)) than OER (*E*>1.4 V vs RHE).[^17^, ^18^, ^19^] With the rise in biodiesel production, there has been a corresponding increase in glycerol waste, creating an opportunity for its transformation. Researchers are actively exploring glycerol oxidation methods, particularly electrocatalytic glycerol oxidation (GOR), as a means to efficiently convert glycerol into valuable organic compounds under mild conditions. GOR, characterized by its lower oxidation potential compared to OER, not only has the potential to reduce overall energy consumption of an electrolysis cell, but also serves a dual purpose. Indeed, it generates valuable high‐value compounds with industrial and pharmaceutical applications, thereby offsetting long‐term costs and showcasing the potential of the glycerol electro‐reforming in alkaline electrolysis cells.[^21^, ^22^] For the organic compounds electrooxidation, the catalytic activity is expected to be maximal at pH close to the compound′s p*K*a,^[26]^ which justifies the study of GOR in alkaline media because glycerol has a p*K*a of 14.4. For such glycerol‐fed electrolyzers, the advancement of energy‐efficient electrolysis systems hinges on the development of active and stable catalysts for GOR and HER. There is substantial room for innovation in both the electrocatalyst design and the optimization of the electrolyzer itself to harness this efficiency. Innovations in electrolyzer′s components such as flow reactors and multifunctional electrodes should simplify the system setup and boost the overall performance.^[26]^ It can be observed that the existing literature is dominated by Pt, Pd and Au based electrocatalysts for efficiency reasons which cannot be achieved with Ni, Fe, Cu, Co, etc.: (i) maintain the anode potential below 1 V vs RHE at a significant current densities (larger than 0.1 A cm^−2^) to minimal the energy input during the electrolyzer operation in real situation, (ii) control the GOR selectivity to the desired products by avoiding side reactions such as OER, complete oxidation of organics, and corrosion of the support. PtAu alloys, in particular can enable to meets these criteria leveraging on electronic interaction between Au and Pt to improve catalytic efficiency, selectivity and durability.^[28]^ The formation of a PtAu alloy changes the interatomic distance, which in return affects the electronic structure of platinum and gold, known as the electronic exchanges between the 5*d* orbitals of gold (10 e^−^) and those of Pt (9 e^−^).^[31]^ Such electronic interactions induce changes in the valence band energies of Pt and Au, thus their catalytic activity. In fact, the binding energy and the catalytic activity of multimetallic catalysts can be fine‐tuned by three fundamental effects, namely, ensemble (synergistic, several metals), ligand (short‐range electronic charge‐transfer effect) and strain (long‐range geometric lattice strain).^[35]^ For bimetallic electrocatalysts, the challenge is to synthesize alloys with a significantly reduced total metal loading for few to tens of μg cm^−2^ onto the electrolyzers’ supports such as gas‐diffusion electrode (GDE), which could enable to bridge the gap between fundamental and applied research.^[38]^ The ultimate target is to keep the key modules (metal loading, support, etc.) unchanged between half‐cell and electrolyzer tests in order to minimize any performance trend discrepancy.^[38]^

We note that the synthesis of multifunctional catalysts for both HER and GOR, particularly the bimetallic nanostructures, demands on many steps for the meticulous control of material characteristics to meet the unique kinetic and thermodynamic requirements of each reaction in the electrolysis process.^[39]^ Traditional methods for preparing PtAu electrocatalysts typically require either chemical or physical reduction of metal precursors, a process frequently requiring surfactants or capping agents. While these substances aid in the particle size control step, they may also impede the exposure of active sites on the catalyst and/or require a much higher metal loading during the real electrolyzer operation.^[21]^ Therefore, there is an open research challenge for innovative PtAu‐based electrocatalyst design methods that precisely adjust the electronic structure and allow decreasing significantly the metal loading to 10–100 μg cm^−2^ thereby enhancing the catalytic performance and applicability in electrolyzers. A possible methodology is using the electroshock approach, which quickly switches the applied bias (potential, current) to overcome any reduction kinetics discrepancy and trigger the formation of relatively uniform nanostructures directly onto the electrocatalysts support such as carbon plates, metal foams, gas diffusion electrodes (GDEs), etc.[^21^, ^40^]

In this study, we tackle the above challenges with a proof‐of‐concept pulsed electrochemical method for the controlled reduction of Au(III) and Pt(IV) directly onto GDE to form ultra‐low loading PtAu nanoalloys (12–26 μg cm^−2^) as multifunctional free‐standing electrocatalysts for a glycerol‐fed electrolyzer. Our galvanostatic electroshock synthesis enables precise deposition of particles onto GDE (GDE‐Au_100–x_Pt_x_, x=0–100, atomic percentage of Pt) for direct assembly of “membrane‐electrode‐assembly, MEA” thus suppressing any testing discrepancy between the half‐cell and electrolyzer experiments.^[38]^ The other advantage is eliminating the need for catalytic ink and thus avoiding binder use during catalytic ink preparation as well as particle agglomeration during catalysis, which diminish the catalyst efficiency. Prior to examining the bimetallic nanostructure′s composition, we investigated the influence of the galvanostatic electroshock parameters (the current and the duration of pulses), achieving promising results in half‐cell configurations. These findings, combined with the refinement of electrolytic media parameters, paved the way for the subsequent exploration of bimetallic nanostructure composition for glycerol‐fed alkaline electrolyzer. A key aspect of our results is the selective electrocatalytic oxidation of glycerol to high‐value organic compounds, for which we conducted detailed product analyses by combining bulk electrolysis and high‐performance liquid chromatography (HPLC). Also, we developed a hybrid electrolyzer where GDE‐Au_100–x_Pt_x_ functions as both anode and cathode. This aims to efficiently and simultaneously electro‐synthesize high value‐added fuels and chemicals, H_2_ at the cathode and organics at the anode with a significantly reduced metal loading of 12–26 μg cm^−2^. The integrated and optimized GDE‐Au_100–x_Pt_x_ (x=30–70) composite significantly enhances the flow electrolyzer′s performance, especially by exhibiting increased electrocatalytic activity and selectivity at the anode in converting glycerol into glycolate and glycerate, in addition to H_2_ at the cathode.

## Experimental Section

### Reagents and Materials

AvCarb MGL190 carbon paper electrodes (GDE, 190 μm thickness) from Fuel Cell Earth LLC (USA) and electroplating tape from 3 M Company were used for GDE‐based experiments. Chemical reagents, including potassium tetrachloroaurate (III) hydrate (KAuCl_4_ ⋅ xH_2_O, 99 %), dihydrogen hexachloroplatinate (IV) hexahydrate (H_2_PtCl_6_ ⋅ 6H_2_O, 99.95 %), potassium bromide (KBr, 99 %), isopropanol (iPrOH, 99.5 %), glycerol (C_3_H_8_O_3_, >99.5 %), and calcium DL‐glycerate dihydrate (C_6_H_12_CaO_9_, >99 %) were sourced from Alfa Aesar. Nafion™ perfluorinated resin suspension and glycolic acid (C_2_H_4_O_3_, 99 %) were acquired from Sigma‐Aldrich. Sodium hydroxide (NaOH, 99 %) were purchased from J. T. Baker. Potassium nitrate (KNO_3_, >99 %), tartaric acid (C_4_H_6_O_6_, >97 %), oxalic acid (C_2_H_2_O_4_, >99 %), and formic acid (CH₂O₂, >99 %) were acquired from Fluka. Hydrochloric acid (HCl, 37 %) and nitric acid (HNO_3_, 68 %) were purchased from VWR International (Fontenay sous Bois, France). Commercial Pt/C (20 wt %, 2 nm particle size) and Au/C (20 wt %, ≈4 nm particle size) were purchased from Premetek Co. (USA) and used as‐received. A hydroxide anion exchange membrane (AEM), Sustainion® X37‐50 Grade RT (50 μm dry thickness) was procured from Fuel Cell Store (USA). The membrane underwent activation in 1 M NaOH for 24 hours at 22±3 °C and was subsequently rinsed abundantly with Milli‐Q water. Nitrogen (grade 4.5) and argon (grade 5.0) gases for the experiments were sourced from Air Liquide, France. High‐purity water (18.2 MΩ cm resistivity at 20 °C) from a Milli‐Q Millipore system and deionized water (>12 MΩ cm resistivity) were used consistently. All reagents and chemicals, of analytical grade, were used as received without further purification. A standardized cleaning method was applied to all glassware used in the electrochemical experiments.^[44]^

### Synthesis of Free‐Standing GDE‐Au_100–x_Pt_x_ Electrodes

To begin, as‐received GDEs, cut into 3 cm×3 cm T‐shapes, were cleaned by shaking in isopropanol three times for 10 minutes each cycle before drying in an oven at 80 °C for one hour. Electroplating tape was applied to restrict electrodeposition to one side of the GDE. An engineered double‐jacket reactor, thoroughly cleaned with aqua regia^[44]^ (2 vol. HCl (37 %) plus 1 vol. HNO_3_ (68 %)) between syntheses, was utilized for electrodeposition at controlled temperature of 25 °C. This setup included a saturated Hg|Hg_2_SO_4_|K_2_SO_4_ reference electrode (MSE, Radiometer), a 25 cm^2^ glassy carbon (Alfa Aesar) as counter electrode, and a 9 cm^2^ GDE serving as the working electrode. The procedure utilized a SP‐150 potentiostat from Biologic Science Instruments. For a typical synthesis of GDE‐Au_50_Pt_50_, the electrodeposition solution was formulated by mixing 190 mL of 100 mM KNO_3_, 5 mL of 1 mM HAuCl_4_, 5 mL of 1 mM H_2_PtCl_6_ (both stock solutions prepared in 100 mM KNO_3_), and 14 mg of KBr. Prior to electrodeposition, nitrogen gas was bubbled through the solution for 30 minutes to remove dissolved oxygen. During deposition, the solution was gently stirred to ensure uniform mixing. Electrodes, at two different durations with three different total electrical charge unchanged (−4.5 mA (30 min), 4.5 mA (60 min), −9 mA (15 min), −9 mA (30 min), −18 mA (7.5 min), and −18 mA (15 min)), were synthesized to study the impact of current density and pulse duration on galvanostatic electrodeposition. The galvanostatic electroshock program consisted of an OFF step at open circuit potential (OCP; I_OFF_=0, t_OFF_=5 s) followed by an ON step (I_ON_=−9 mA, t_ON_=5 s). For example, this sequence was repeated for a total of 180 times to synthesize GDE‐Au_50_Pt_50_ (−9 mA (30 min)) electrode. By varying the individual volumes of the Au(III) and Pt(IV) stock solutions while maintaining a total combined volume of 10 mL, a library of electrodes with different metal compositions GDE‐Au_100–x_Pt_x_ (GDE‐Au_100_Pt_0_, GDE‐Au_85_Pt_15_, GDE‐Au_70_Pt_30_, GDE‐Au_50_Pt_50_, GDE‐Au_30_Pt_70_, GDE‐Au_15_Pt_85_, and GDE‐Au_0_Pt_100_) were prepared for further investigation. These atomic percentages are theoretical; experimental values have been determined by quantitative elemental analysis (inductively coupled plasma optical emission spectrometry, ICP‐OES, *vide infra*). Thus any normalization by metal mass will be based on experimental values. As Au : Pt atomic ratios differ between theoretical and experimental values, we have not renamed the different materials, so that readers can appreciate the fairness of our approach. After electrodeposition, the final electrodes were rinsed with water and dried overnight at 50 °C. The electroplating tape was then carefully removed. To the best of our knowledge, this is the first time that such a galvanostatic shock program has been developed for GDE‐Au_100–x_Pt_x_ electrodes. To test the commercial catalysts (20 wt % Au/C and 20 wt % Pt/C), catalytic inks were prepared by ultrasonically mixing 1.3 mL MQ water, 0.5 mL iPrOH, 0.2 mL Nafion® suspension and 1 mg catalyst for 30 minutes. The homogeneous ink was drop‐casted onto each face of a bare L‐shape GDE of similar size (as GDE‐Au_x_Pt_y_) and dried at room temperature, achieving loadings of 24.6 μg_Au_ cm^−2^ for Au/C and 0.9 μg_Pt_ cm^−2^ for Pt/C, matching the experimental loadings (by ICP‐OES, *vide infra*) of our synthesized electrocatalysts for fair performance comparison.

### Physico‐Chemical Characterization

Scanning electron microscopy (SEM) was employed to characterize the morphological evolution of Au and Pt nanostructures, utilizing a Hitachi S4800 microscope. Elemental mapping was ascertained through energy‐dispersive X‐ray (EDX) analysis, conducted on a ZEISS EVOHD 15 microscope. The crystalline structure of the grown particles was studied using X‐ray powder diffraction (XRD) analysis conducted on a Bruker D8 Discover diffractometer. This instrument features a Johansson monochromatic optimized for CuKα_1_ radiation and is equipped with a LynxEye XE−T detector, known for its high energy resolution. The diffractometer, set at 40 kV and 40 mA, was used to obtain diffraction patterns in the range of 35° to 85°. These measurements were taken at a rate of 3.6° per minute and with a precise step size of 0.025°. Elemental quantification was performed using inductively coupled plasma optical emission spectrometry (ICP‐OES) on an Agilent 5110 VDV spectrometer. To assess the electronic states and chemical compositions within the synthesized electrodes, X‐ray photoelectron spectroscopy (XPS) was carried out using a Thermo Electron ESCALAB 250 spectrometer (15 kV, 6 mA), operating with monochromatic Al Kα source (1486.6 eV). The spectra are calibrated against Au 4f_7/2_ at 83.96 eV. Spectra were recorded at a 90° take‐off angle, with an analyzed area of approximately 0.7×0.3 mm. Survey spectra were acquired with a 1.0 eV step size and 160 eV analyzer pass energy, while high‐resolution spectra were obtained with a 0.1 eV step size (0.05 eV for O 1 s and C 1 s) and 20 eV pass energy. A neutralizer was employed to counteract charge effects. Data analysis involved fitting curves using Gaussian/Lorentzian (70/30) peak shapes after Shirley background subtraction, performed with CasaXPS software and the quantification was based on peak area after correction by suitable sensitivity factors.

### Electrochemical Characterization in Half‐Cell

All the electrochemical characterization was conducted using a SP‐150 potentiostat (Biologic Science Instruments). The electrocatalytic performance of GDE‐Au_100–x_Pt_x_ electrodes towards GOR and HER were investigated using a conventional three‐electrode cell (single compartment). The cell comprised GDE‐Au_100–x_Pt_x_ (0.5 cm by 0.5 cm of geometric surface area) as the working electrode, glassy carbon with a geometric surface area of 5.5 cm^2^ as the counter electrode, and Hg|HgO|NaOH 1 M (mercury‐mercury oxide electrode, MOE, RE‐61AP, BAS Inc.) serving as the reference electrode. Experiments were conducted in a 1 M NaOH solution saturated with Ar, in the presence of 0.1 M glycerol for GOR, at 25 °C. For electrode activation and stabilization, cyclic voltammetry (CV) was carried out for 15 cycles at a scan rate of 100 mV s^−1^. The HER evaluation was conducted through linear sweep voltammetry (LSV) at a scan rate of 5 mV s^−1^. Electrochemical impedance spectroscopy (EIS) was performed over a frequency range from 100 kHz to 100 mHz at different potentials. All reported potentials in this study are converted to the RHE scale for consistency and comparison. To convert the potentials to RHE scale, we employed the formula (*E*_(RHE)_=*E*_(MOE)_+0.92), the calibration curve, relative to the MOE scale, is provided in Figure S1. The potentials are ohmic‐drop uncorrected (ohmic resistance was about 4 Ω, 1 M NaOH and 0.5 cm^2^ GDE).

### Electrolysis in Hydroxide AEM Based H‐Type Cell and High‐Performance Liquid Chromatography (HPLC)

Bulk electrolysis for GOR conversion and product distributions was carried out at 25 °C in a temperature‐controlled H‐type cell (two‐compartment). The working electrode compartment, housing a 5 cm^2^ GDE‐Au_100–x_Pt_x_ as working electrode and MOE as reference electrode, contained 1 M NaOH and 0.1 M glycerol (35 mL). The counter electrode compartment, equipped with a 17.5 cm^2^ glassy carbon slab, held only 1 M NaOH (35 mL). The two compartments were separated by the hydroxide AEM (Sustainion® X37‐50 Grade RT). Electrolysis was conducted by two different methods, chronoamperometry (CA) at an applied potential *E*_applied_=0.89 V vs RHE and chronopotentiometry (CP) at an applied current density *j*_appied_=20 mA cm^−2^, for a duration of 1 hour at a temperature of 50 °C. The final products, resulting from glycerol electrooxidation, identification and quantification were achieved via HPLC using a Dionex ICS‐1000 system. Organic acids were separated by p*K*a strength using an UV‐vis detector (λ=210 nm) on a BP‐OA Benson 2000–0 column, with an injection volume of 25 μL and an eluent of 25 mM H_2_SO_4_ at a flow rate of 0.4 mL min^−1^. This process involved chromatographic separation based on retention time and quantification via peak area analysis. Calibration curves for expected standard reactants–including oxalic acid, tartronic acid, glyceric acid, glycolic acid, and formic acid–was established, ranging from 0.025 mM to 2 mM, with linear regression R^2^ values exceeding 0.99. To maintain the stationary phase of the column, samples were diluted with ultrapure water to reduce their alkalinity.

### Hydroxide AEM Based Electrolyzer and H_2_ Collection

Hydroxide AEM‐based electrolyzers were utilized for biomass‐based electrolysis experiments. A detailed schematic of the electrolyzer setup can be found in Figures S2–S3. The assembly consists of end plates, current collector plates, diffusion layers, flow plates, and Teflon gaskets from Scribner, LLC (USA). Heating elements and a thermocouple embedded within the end plates enabled temperature control (set at 50 °C). The electrolysis cell features a total active surface area of 5 cm^2^. Pairs of GDE‐Au_100–x_Pt_x_ electrodes, which serve as multifunctional electrocatalysts for both HER and GOR, were separated by the AEM. The assembled cell was then mechanically compressed and set at 50 °C. Operating conditions were set at a catholyte circulation rate of 45 mL min^−1^ for 1 M NaOH and an anolyte circulation rate of 23 mL min^−1^ for a solution containing 1 M NaOH+1 M glycerol, driven by gear pumps. The recirculation solutions were kept at a water bath at 50 °C. Electrochemical performance was evaluated by two different methods: LSV (with a scan rate of 0.05 V s^−1^) and potentiostatic (potential increments of 0.1 V for different dwell times). The employed potentiostat featured a 10 A/20 V booster (VMP3B‐10, Biologic Science Instruments), facilitating the application or recording of higher currents and voltages. The water displacement method was employed to measure generated hydrogen as illustrated in Figure S2.^[45]^ When the current was fixed (100 mA, 20 mA cm^−2^ so that all electrodes could be compared), H_2_ was collected, thus allowing for the quantification of hydrogen production in real‐time. The determined hydrogen flow rate was deduced and subsequently compared with the theoretically calculated flow rate (Faraday law).

## Results and Discussion

### Role of the Current Density and the Duration of Pulsed Galvanostatic Electrodeposition

**Figure 1:**
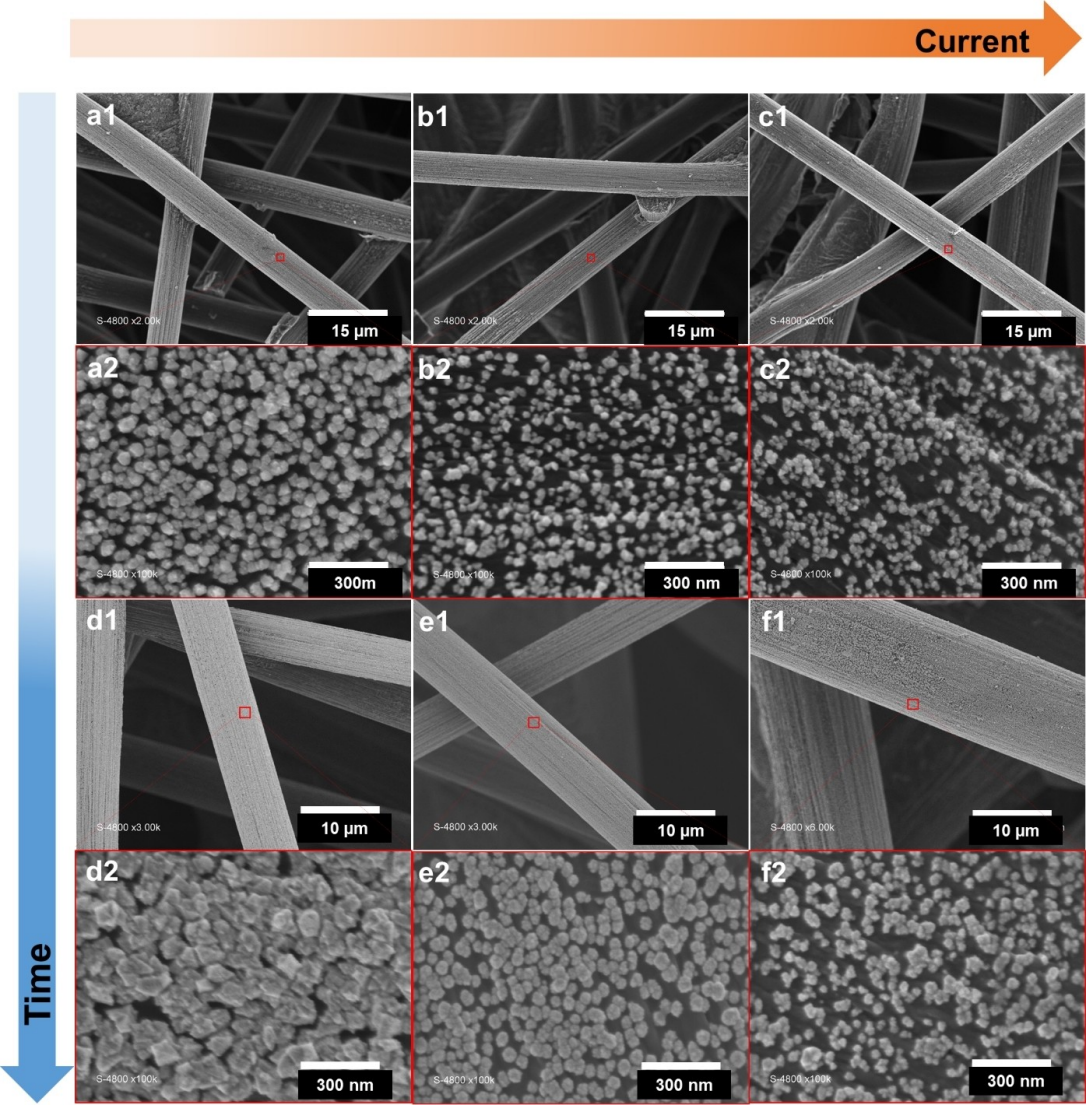
SEM images of Au_50_Pt_50_ (theoretical; experimental values are reported in Tables S1–S2) grown on a one face of GDE (3 cm by 3 cm) for different duration and current using pulsed galvanostatic electrodeposition: a1–a2) −4.5 mA (30 min), b1–b2) −9 mA (15 min), c1–c2) −18 mA (7.5 min), d1–d2) −4.5 mA (60 min), e1–e2) −9 mA (30 min) and f1–f2) −18 mA (15 min).

Conventional electrodeposition is often constrained by the sluggish migration of metal ions to the substrate. To circumvent this limitation, our study employed pulsed galvanostatic electrodeposition, an approach gaining recent attention for its utility in nanoparticles synthesis.^[47]^ These pulsed techniques offer advantages over conventional galvanostatic methods, including improved grain morphology and enhanced structural integrity of the deposited noble metals. In our approach, the nucleation of nanoparticles is initiated by a pulse of constant current, which leads to a rapid decrease in metal ion concentrations near the electrode due to rapid electrochemical reactions. This step is followed by a relaxation period, allowing ions to diffuse to the substrate for subsequent reduction.[^40^, ^49^] Systematic variations in experimental conditions, including reaction time and current density, were employed to elucidate the growth kinetics of Au and Pt nanoparticles via electrodeposition. We have previously demonstrated the impact of adding KBr in different molar ratio of n(KBr)/n(metal) on the growth kinetics.^[50]^ The KBr effect can be attributed to the enhanced stability of the [AuBr_4_]^−^ and [PtBr_6_]^2−^ species (in comparison to the chloride forms). These species exhibit less susceptibility to dissociation, likely due to their higher p*K*_d_ values, indicating a stronger bond formation in the presence of bromide ions.^[51]^ In the electrodeposition process of metals and alloys, the surface morphology is shaped by multiple phenomena. These include the diffusion and movement of ions from the electrolyte to the electrode, and the processes of nucleation and surface growth.^[52]^ To track the changes in particle morphology and size of nanoparticles, SEM analysis was utilized, as depicted in Figure 1a1–f2. SEM‐EDX maps are reported in Figure S4 (quantitative data in Table S1). The nominal atomic composition Au_50_Pt_50_ was chosen to optimize the electrodeposition parameters for a better compromise between the reduction kinetics of Au(III) and Pt(IV) to have Au and Pt on the one hand, and the concomitant HER during metal deposition on the other. During electroshock synthesis (pulse electrodeposition), the first deposited metallic particles catalyze HER, whereby the rapid generation and dissipation of H_2_ bubbles partially inhibits the coalescence and the fusion of seeds into bigger particles.[^41^, ^42^, ^43^, ^53^]

For a 3D substrate such as GDE with anisotropic surface (as opposed to flat glassy carbon electrodes), we hypothesize that the magnitude of the current and duration of reaction could be effective tools to regulate the nucleation and growth of gold and platinum seeds into particles of optimized sizes, which is very important for better electrocatalysis. Figure S5 shows the size distribution of the nanoparticles. When the only variable was the current density, a reduction in particle size was noted (for example Figures 1a to 1c). This can be attributed to an enhanced nucleation rate of seeds and high amount of H_2_ bubbles that partially inhibits the coalescence and the fusion of seeds into bigger particles,[^41^, ^42^, ^43^, ^53^, ^54^] which results in smaller particles and a greater number of active sites per unit area.^[52]^ Consequently, metal ions are distributed across a larger number of growing particles. Conversely, prolonging the reaction time resulted in an increase in particle size, as evidenced by the microscopic data (for example Figures 1a and 1d). This enlargement is probably due to the electrode′s relaxation time, which promotes effective ion diffusion and supports continuous growth of the particles.^[55]^

To gain more insights into the electrochemical and electrocatalytic properties of the Au−Pt particles deposited onto GDE, we conducted a comprehensive series of tests. The overarching goal was not merely to delineate the optimized parameters for pulsed electrodeposition but also to find the best compromise between the microstructure and the ability to be used as multifunctional electrodes for glycerol‐fed hydroxide AEM‐based electrolyzers. Thus, the electrodes were used for both GOR and HER in an alkaline medium. The CV profiles in 1 M NaOH (Figure S6a) delineate the electrochemical characteristics of Au−Pt alloy in an alkaline electrolyte.^[33]^ Figure S6b shows the forward scan of the CV in the presence of glycerol where the onset potential is ca. 0.2 V vs RHE. The increase of the current density for electrodes synthesized by a prolonged electrodeposition program (Figure S6b) is a proof of an enhancement of the electrochemical kinetics. Increasing the electrodeposition time or decreasing the current favors more metal deposition on GDE, yet this is accompanied by an increase in electronic exchanges between Au and Pt (a partial electrons transfer from 5*d* ^10^ orbitals of gold to those of Pt (5*d* ^9^), resulting in greater electrocatalytic efficacy.[^31^, ^33^, ^34^] Specifically, at 0.8 V vs RHE, the highest current density for GOR is 27.0 mA cm^−2^ for 30 min of deposition time (15 min ON and 15 min OFF) at −9.0 mA, seconded by 60 min of deposition time at −4.5 mA (15.4 mA cm^−2^ for GOR). This improvement in the electrocatalytic kinetics is further evidenced by the reduced overpotential for the HER as shown in Figure S6c. The catalytic activity towards GOR is influenced by the size of the nanoparticles. Smaller nanoparticles provide a higher surface area to volume ratio, which enhances the availability of active sites for the reaction. However, extremely small nanoparticles may lead to increased surface energy, causing agglomeration and loss of catalytic activity (Figure S5 and S6). In our study, nanoparticles with an average size of 45–50 nm showed the highest catalytic activity for GOR, as evidenced by the current densities observed in Figures 3a and 3b. This optimal size balances the need for a high surface area with the stability of the nanoparticles. For HER, larger nanoparticles demonstrated superior performance. HER involves a 2‐electron process at the cathode, which is not as kinetically limited as OER. Studies have shown that HER activity on single‐crystal surfaces follows the trend (111)<(100)<(110), due to decreases in activation energy.^[57]^ Larger nanoparticles expose more high‐activity facets (e. g., (110) facets), enhancing HER performance. Additionally, they exhibit lower surface energy and increased stability, reducing particle aggregation. The combination of lower onset potentials, increased current density, decreased overpotentials, and lower charge‐transfer resistances (see Figure S6d), highlights the effectiveness of this method in creating multifunctional catalysts for both GOR and HER. These results strongly support our hypothesis regarding the impact of electrodeposition parameters on the electrocatalytic efficiency, illustrating the significance of meticulously controlled galvanostatic electroshock synthesis conditions in refining the multifunctional electrocatalytic properties essential for AEM‐based electrolyzers. Based on HER and GOR performance, the electrodeposition conditions were a current of −9 mA and a duration of 30 min to study impact of bimetallic composition.

**Figure 2:**
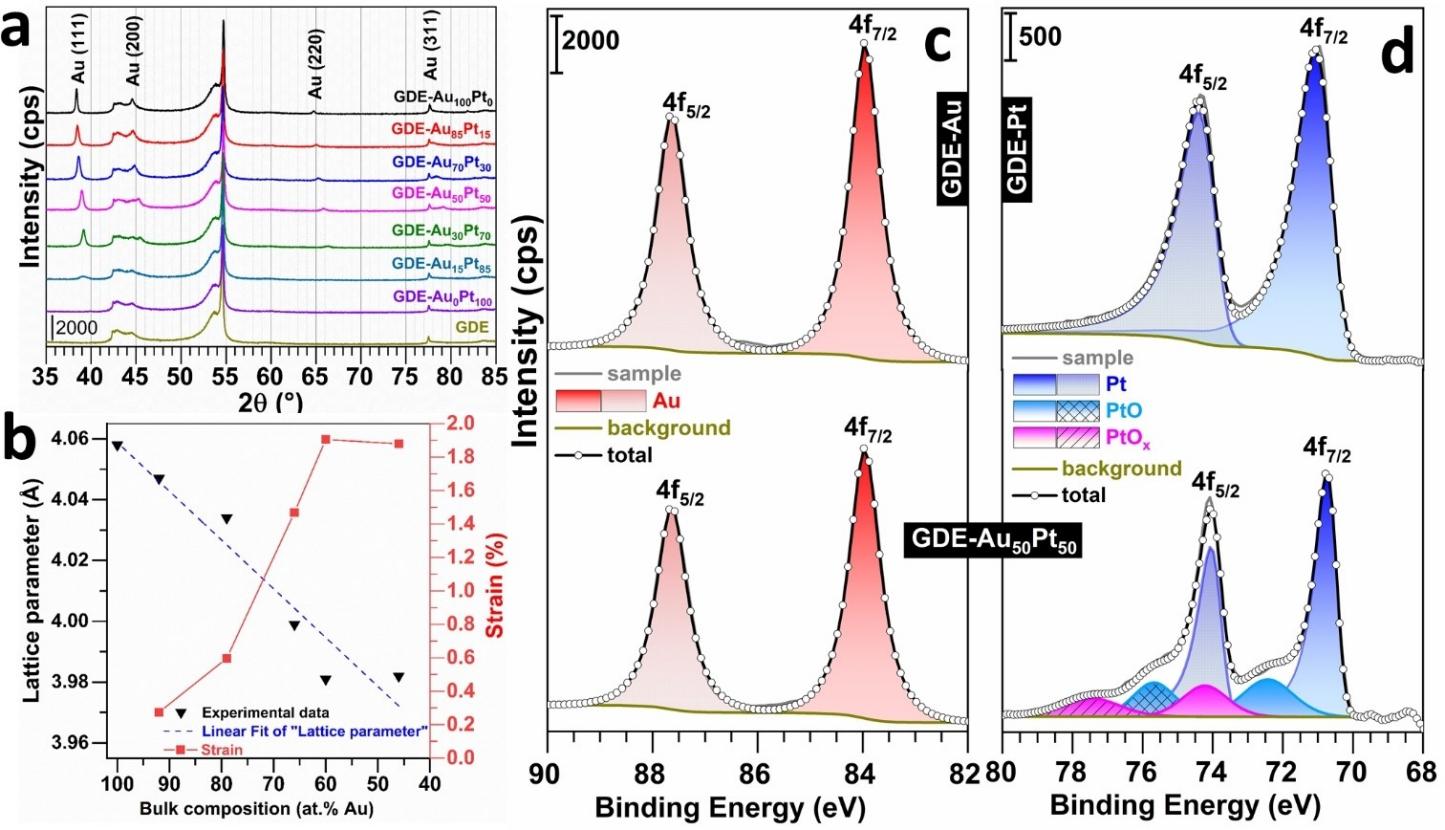
a) XRD patterns of the GDE‐Au_100–x_Pt_x_. b) Influence of incorporation of Pt atoms into Au nanoparticles (and vice versa) on crystal lattice parameter of the GDE‐Au_100–x_Pt_x_ material. c–d) High‐resolution XPS spectra of the GDE‐Au_100–x_Pt_x_ material: c) Au4f XPS spectra of GDE‐Au_100_Pt_0_ (top) and GDE‐Au_50_Pt_50_ (bottom, experimental Au : Pt is 64 : 36, Table S4), and d) Pt 4f XPS spectra of GDE‐Au_0_Pt_100_ (top) and GDE‐Au_50_Pt_50_ (bottom). Note: GDE‐Au_X_Pt_y_ refers to as theoretical composition and the true experimental “bulk atomic composition” was determined from ICP‐OES and gathered in Table S4.

**Figure 3:**
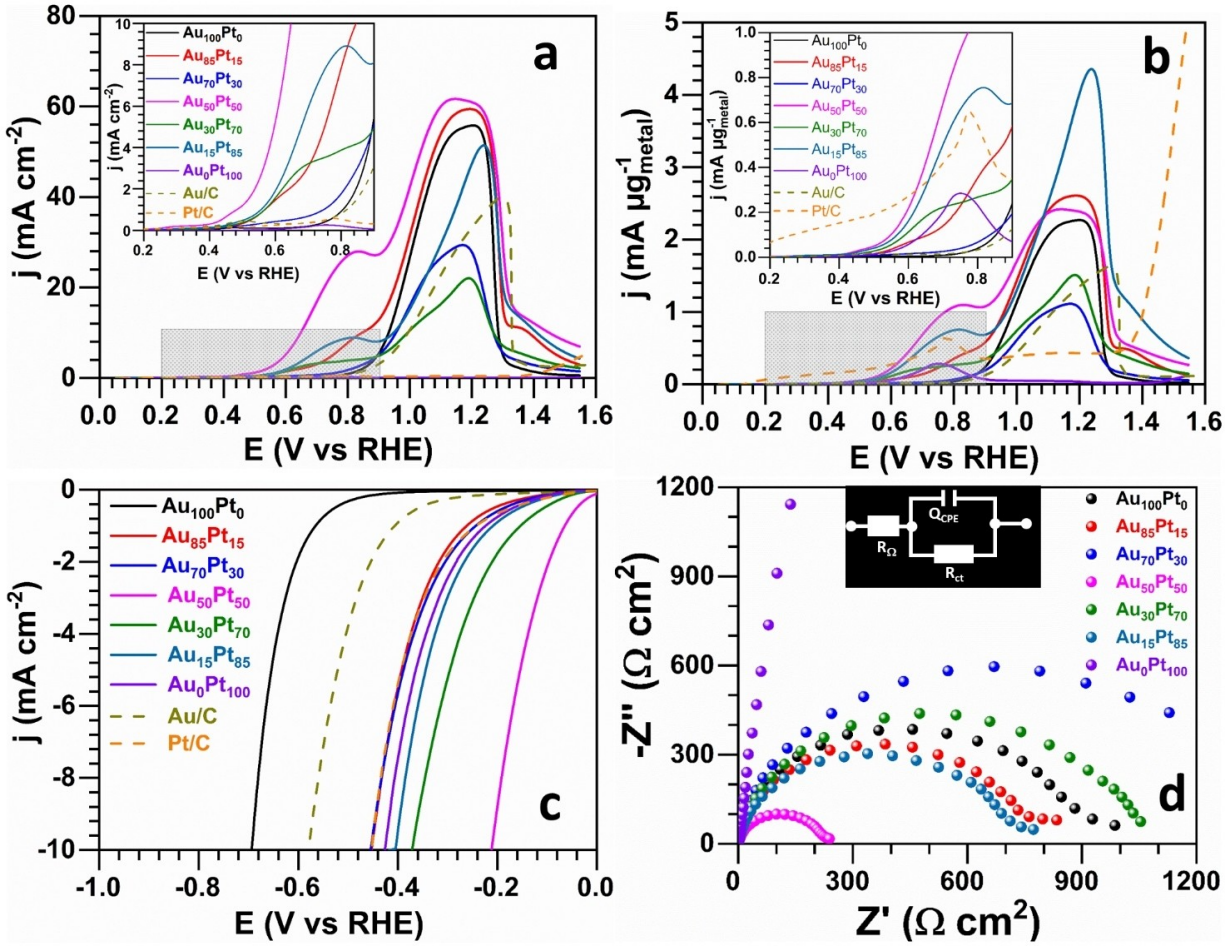
Electrochemical performance of the as‐synthesized GDE‐Au_100–x_Pt_x_ and the commercial electrocatalysts (Pt/C and Au/C). a–b) Forward scan of the CV of GOR (50 mV s^−1^, 0.1 M glycerol, 1 M NaOH, 25 °C); current density normalized by: a) estimated geometric surface area and b) the experimental (total) metals content determined from ICP‐OES (Table S4). c) LSV curves of HER (5 mV s^−1^, 1 M NaOH, 25 °C) and d) Nyquist impedance plots at −0.42 V vs RHE for HER. Potentials are iR‐drop uncorrected. Note: GDE‐Au_X_Pt_y_ refers to as theoretical composition and the true experimental “bulk atomic composition” was determined from ICP‐OES and gathered in Table S4.

### Effect of the Composition of the Bimetallic Nanostructures

To understand the role of the composition and synergistic effects of electrochemically incorporated Au and Pt onto GDE, the electrodes with the most favorable particle distribution across the electrode surface and the best performance during HER and GOR from our previous investigation were used as optimized conditions, namely the I_ON_=−9 mA and ∑(t_ON_+t_OFF_)=30 min. A comprehensive range of Au−Pt compositions was investigated, spanning from pure gold (GDE‐Au_100_Pt_0_) to pure platinum (GDE‐Au_0_Pt_100_). The SEM and EDX mapping presented in Figures S7–S8, which confirm the successful and uniform decoration of the GDE surface with Au and Pt across all samples. Table S2 gathers the qualitative analysis of the chemical composition. In bimetallic materials, the experimental atomic percentage of Au is a bit higher due to the discrepancy in higher redox potentials associated to the Au(III)/Au and Pt(IV)/Pt, specifically, *E*°(AuBr_4_^−^/Au)=1.0 V vs SHE compared to *E*°(PtBr_6_^2−^/Pt)=0.85 V vs SHE.^[58]^ This could also be explained by the superior kinetics of Pt for HER compared to Au when the first seeds are formed from the applied current bias, which also translates into a decrease in particle size as the Pt content increases. Moreover, while the used GDE substrate consists of ca. 30 layers of microfibers (thickness of 190 μm and an average fiber diameter of 7 μm), particles are deposited only on the first 2–3 layers of microfibers, irrespective of the initial wetting of the electrode by the electroplating solution. This has been observed previously[^21^, ^50^] and can be explained by a shielding effect. The outer microfibers are much closer to the counter‐electrode, and are therefore subjected to a higher local electric field between the working electrode and the counter‐electrode, resulting in preferential nucleation and growth on the outer microfibers. Not only does this establish a concentration gradient for the metal precursor, but it also creates a shielding effect that could promote diffusion of the metal precursor towards the area where the first seeds are produced, that is, the outer microfibers.

We next used XRD to determine the crystallographic structure of the fabricated electrodes. As depicted in Figure 2a, the blank GDE exhibited a significant peak at 54.7°, corresponding to the (004) reflection peak of graphite, a characteristic of the carbon support.^[60]^ This peak was consistently observed across all synthesized GDE‐Au_100–x_Pt_x_ electrodes. For the GDE‐Au_100_Pt_0_ sample, the presence of peaks at 38.3°, 44.6°, 64.7°, and 77.7° correspond to the (111), (200), (220), and (311) crystallographic orientations of face‐centered cubic (fcc) gold. In electrodes featuring varied ratios of Au and Pt, the XRD patterns exhibited shifts in peak positions and variations in peak intensities. These observations indicate changes in the crystallographic structure due to Pt incorporation. In the GDE‐Au_0_Pt_100_ pattern, the Pt peak was too weak to be distinctly observed (because of a low Pt content, 1 μg_Pt_ cm^−2^). The quantitative data are reported in Table S3. The XRD‐derived graph in Figure 2b of Vegard′s‐law‐based plots^[33]^ highlights the decreasing of the lattice parameter against the increasing atomic percentage of platinum, indicative of lattice contraction with platinum integration into the gold structure. The corresponding strain,[^35^, ^61^] increases from 0.3 % to 1.9 % as platinum content rises, suggests lattice stress due to the size mismatch between gold and platinum atoms. Notably, the strain levels off at higher platinum contents, implying a limit to lattice distortion. The data thus implies a specific compositional range for the optimal microstructure modification, which could be key for enhancing electrocatalytic performance.[^35^, ^61^, ^64^]

To obtain in‐depth knowledge about compositional features of bimetallic material, and surface state of nanocatalysts, we next performed deeper analysis by XPS. The survey XPS spectra shown in Figure S9 reveal distinct features of the Au 4f and Pt 4f regions for GDE‐Au_100_Pt_0_, GDE‐Au_0_Pt_100_ and GDE‐Au_50_Pt_50_ electrodes. Figures 2c–d show the high‐resolution XPS spectra. For the GDE‐Au_100_Pt_0_, the Au 4f region displays characteristic peaks at binding energies of 83.9 and 87.6 eV, which are ascribed to the Au 4f_7/2_ and Au 4f_5/2_ levels, respectively. These peaks confirm the presence of metallic gold on the electrode surface. In the case of the GDE‐Au_0_Pt_100_ spectrum, the Pt 4f region shows peaks at 70.8 eV and 74.1 eV, corresponding to the Pt 4f_7/2_ and Pt 4f_5/2_ levels, indicative of metallic platinum.^[65]^ However, the peaks appear with less intensity in comparison with Au, which may suggest a thinner deposition of platinum or a more dispersed distribution on the electrode surface. Additionally, these facts manifest the efficient reduction of the precursors Au(III) and Pt(IV).^[66]^ The XPS spectra of the bimetallic GDE‐Au_50_Pt_50_ electrodes display a combination of these features, with both gold and platinum peaks present. The binding energy of Pt 4f_7/2_ shifts negatively to 70.5 eV (that of Au 4f_7/2_ was poised at 83.96 eV as reference during XPS calibration), which indicates the formation of a bimetallic alloy and suggest a modification of the electronic structure and charge transfer dynamics between Au and Pt due.[^30^, ^34^, ^39^, ^65^] This results confirm the above microstructure modification as assessed by the lattice strain of 1.5 %. We note that the presence of gold increases the amount of oxidized surface platinum species, which is in agreement with previous observations, probably attributed to weakening of the Pt−Pt bond.^[67]^ These changes have implications for the electrocatalytic performance, particularly for GOR and HER. Table S4 shows the bulk elemental analysis by ICP‐OES, indicating, for monometallic‐based electrodes, gold deposition onto GDE (GDE‐Au_100_Pt_0_, 24.6 μg cm^−2^, 0.27 wt %) and platinum deposition onto GDE (GDE‐Au_0_Pt_100_, 0.9 μg cm^−2^, 0.01 wt %). These results therefore confirm those of XRD, where the Pt signal was not visible, masked by that of carbon. The experimental composition of the bimetallic samples from bulk analysis by ICP‐OES is different from theoretical target of Au : Pt=15 : 85, 30 : 70, 50 : 50, 70 : 30 and 85 : 15 (experimental values of 46 : 54, 56 : 44, 64 : 36, 79 : 21, 92 : 8), with GDE‐Au_50_Pt_50_ showing an almost equal atomic distribution. As previously discussed, the discrepancy between the experimental and theoretical atomic percentages is due to the difference in redox potentials (*E*°(AuBr_4_^−^/Au)=1.0 V vs SHE, *E*°(PtBr_6_^2−^/Pt)=0.85 V vs SHE)[^58^, ^68^] and the superior kinetics of Pt for HER compared to Au as soon as the first seeds are formed from the applied current bias.

We next broadened our study to encompass a comparative analysis of how bimetallic compositions affect electrocatalytic behavior. Commercial Au/C (20 wt %) and Pt/C (20 wt %) nanocatalysts served as benchmark materials to ensure a fair and standardized comparison. The results are reported in Figures 3a–f for both GOR and HER. Table S4 gathers EIS data of HER. The blank CVs in 1 M NaOH electrolyte of Figure S10 confirm the presence of Pt and Au for the various electrodes.[^33^, ^67^] The GDE‐Au_100–x_Pt_x_ electrodes were first subjected to the electrocatalytic test toward GOR. The comparison with relevant metallic electrocatalysts for GOR in alkaline media is summarized in Table S5. Forward scan CV results for GOR (Figure 3a and b) illustrate a notable influence of the bimetallic interaction between Pt and Au on the electrocatalytic activity towards GOR. Across the range of bimetallic compositions tested, there is a negative shift in onset potentials and an increase in current densities when compared to the pure GDE−Pt and GDE−Au samples. This data indicates that the bimetallic electrodes facilitate GOR at lower potentials than their pure counterparts, as less energy is required to initiate glycerol oxidation, demonstrating enhanced kinetic ability and catalytic efficiency. Specifically, some bimetallic ratios yield greater current densities than the pure Au sample, highlighting the synergistic effect of combining Au and Pt. These results suggest that the electrocatalytic activity is not solely dependent on the content of Au but rather on the optimal interplay between Pt and Au within the bimetallic structure. The improved activity of these bimetallic catalysts could be due to a more favorable electronic structure for GOR, leading to a more effective facilitation of the reaction at the catalytic sites.[^31^, ^32^, ^34^, ^69^] The improvement might be due to glycerol molecules reacting on the more active Au−Pt interface, coupled with the bifunctional effect, whereby Pt provides adsorbed hydroxyl species for the reaction on Au at lower potentials.^[30]^ This suggests that the bimetallic nature of the catalysts enhances electrocatalytic activity, influencing the efficiency of glycerol oxidation irrespective of the specific Au−Pt ratios. It worth to mention that balanced ratio of metals (GDE‐Au_50_Pt_50_, which is actually 64 at . % Au and 36 at . % Pt) shows the most substantial improvement for GOR, reaching a record peak current density of 2.5 A mg^−1^ (59.7 mA cm^−2^).^[70]^ To further illustrate the intrinsic catalytic activity, the performance of GDE‐Au_100_Pt_0_ and GDE‐Au_0_Pt_100_ electrodes was compared with that of commercial Pt and Au catalysts (Figure 3). The comparative analysis highlights that electrodeposited Au and Pt surpass commercial electrodes, with improvements in onset potential and marked enhancements in catalytic current for GOR. We know that Au−Pt particles are only deposited on the first 3 microfiber layers, whereas in the case of catalytic inks derived from Pt/C and Au/C, all microfiber layers (around 30) should contain Pt or Au. Thus, normalization by the number of microfiber layers actually containing catalytic particles results in a substantial increase in activity that can reasonably be attributed to the synthesis method, since direct contact of the active sites with the GDE support accelerates electron transfer kinetics. These findings underscore the effectiveness of electrodeposition in advancing the performance of these metals beyond that of standard electrocatalysts.

**Figure 4:**
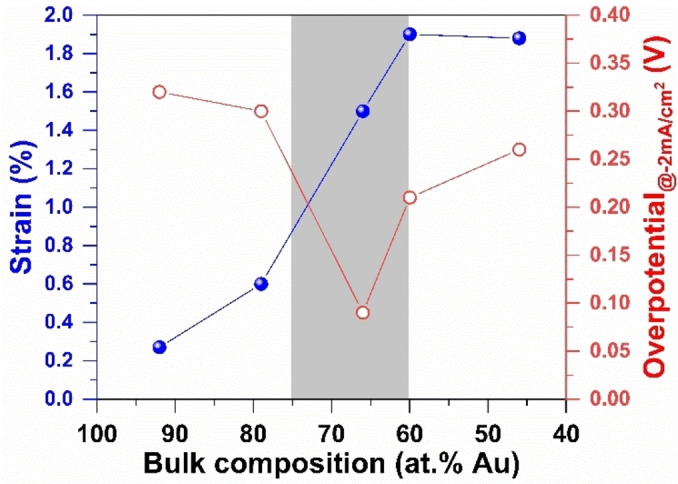
Strain‐dependent HER overpotentials (from LSV curves at 5 mV s^−1^ in 1 M NaOH at 25 °C). Experimental “bulk atomic composition” was determined from ICP‐OES (Table S4).

We next explored the HER activity of our electrodes, using the current density (j) at −10 mA cm^−2^ as the standard metric for examining and comparing the electrocatalytic HER activity of various electrocatalysts.^[75]^ The LSV curves in Figure 3c highlight that most of bimetallic GDE‐Au_100–x_Pt_x_ electrodes outperform the monometallic GDE−Pt and GDE−Au electrodes, with a significant decrease in overpotential needed to achieve this benchmark current density (Table S6). The trend indicates that as the Pt content in bimetallic electrodes increases, the overpotential decreases, suggesting Pt′s pivotal role in enhancing HER activity. Notably, electrodes with a balanced Au−Pt ratio exhibited the lowest overpotential (Figure 4). Similar to GOR, this optimal performance can be attributed to the electronic effect caused by a shift in the d‐band center of Pt in AuPt alloys, which serves as a potent electrocatalytic platform for the HER. The synergistic effect of the bimetallic composition underlines the importance of electronic structure modifications in achieving efficient electrocatalysis. EIS analyses (Figure 3d) further support these findings, where lower charge‐transfer resistances correspond to the sample with balanced metal ratio, signifying an improved electron transfer process which is essential for efficient catalysis (EIS fitted data are reported in Table S4). These results collectively demonstrate the pivotal role of bimetallic composition in optimizing electrocatalytic behavior. The ability to tune the electrochemical properties by varying the Pt to Au ratio presents a significant opportunity to enhance the performance of electrodeposited GDE‐AuPt electrodes in both GOR and HER, crucial for the advancement of biomass‐fed hydroxide AEM‐based electrolyzers.

### Impact of the Method of Bulk Electrolysis (Hydroxide AEM Based H‐Type Cell) on the Selectivity of GOR

The primary aim of this study segment was to evaluate if different techniques used in recording polarization curves for bulk electrolysis could lead to variations in the distribution of glycerol oxidation products (see Figures S11–S14). This analysis is vital for comprehending the kinetics and mechanism of the electrocatalytic process. To this end, we employed two distinct electrochemical techniques for bulk electrolysis: chronoamperometry (CA, potentiostatic electrolysis) and chronopotentiometry (CP, galvanostatic electrolysis). CA allows for the maintenance of a constant potential over time, thereby providing insights into how glycerol oxidation products composition evolves under steady‐state conditions. In contrast, CP keeps the current constant while monitoring the evolving potential, offering complementary insights into the dynamic behavior of electrocatalysts under different operating conditions.^[79]^ The setup of bulk electrolysis, characterized by larger electrode surface areas, solution stirring, and specific electrode separation, significantly influences the selectivity of GOR.^[80]^ We note that bulk electrolysis in an H‐type cell offers a comprehensive and uniform oxidation environment, which differs markedly from the constrained electrolysis in voltammetry cells. This difference, combined with the contrasting approaches of CA and CP, enables a thorough understanding of GOR.

**Figure 5:**
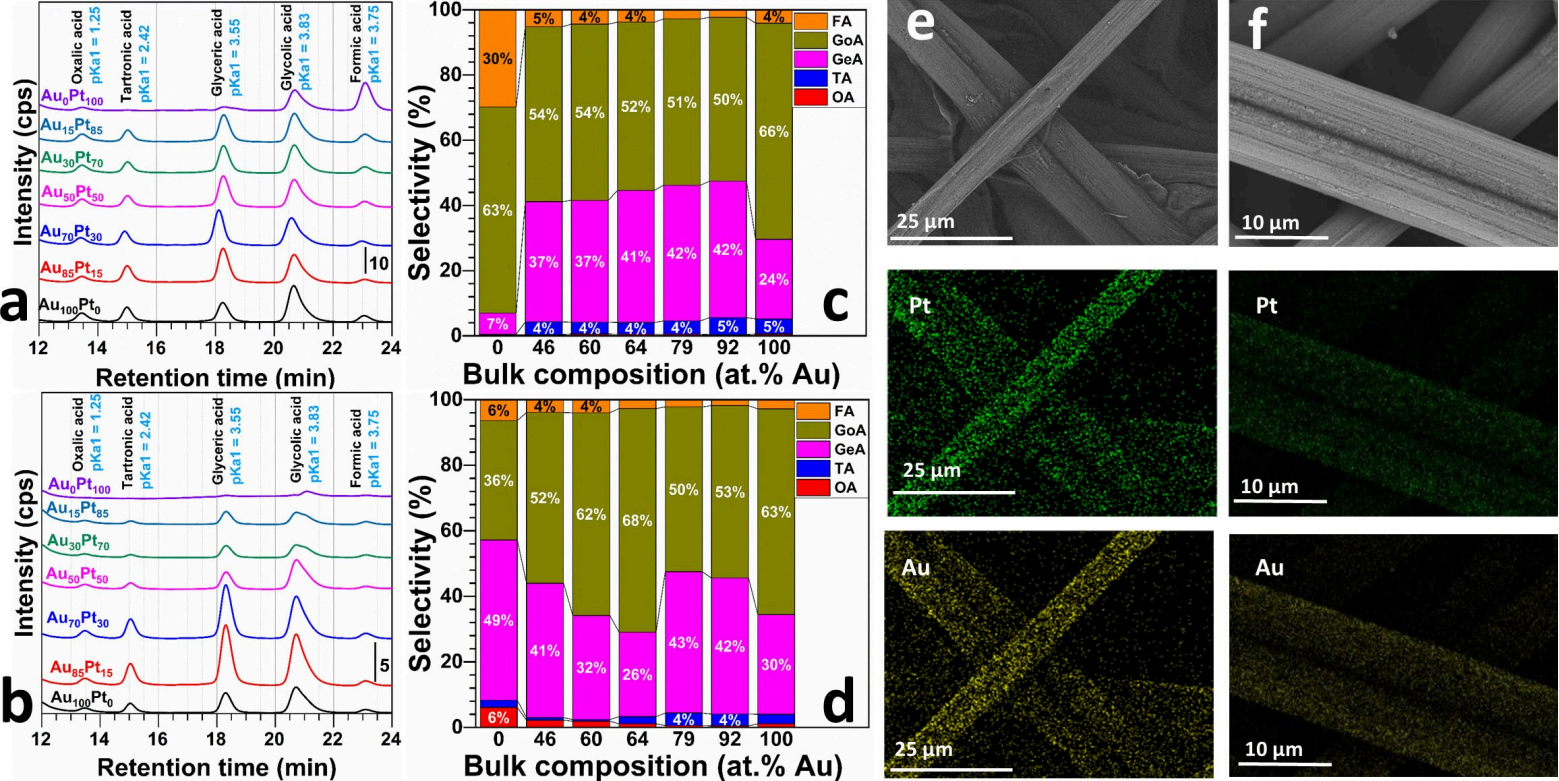
a–d) The products distribution chromatograms and relative bar charts from HPLC analysis: a and c) after 1 hour glycerol electrooxidation (1 M glycerol) using CA methodology (E_apply_=0.89 V vs RHE, 1 M NaOH, 50 °C), b and d) after 1 hour glycerol electrooxidation (1 M glycerol) using CP methodology (j_apply_=20 mA cm^−2^, 1 M NaOH, 50 °C). e) SEM‐EDX mapping of GDE‐Au_50_Pt_50_ and f) Post‐mortem SEM‐EDX mapping of GDE‐Au_50_Pt_50_. Key: FA: Formic Acid, GoA: Glycolic Acid, GeA: Glyceric Acid, TA: Tartronic Acid, OA: Oxalic Acid. Note: GDE‐Au_X_Pt_y_ refers to as theoretical composition and the true experimental “bulk atomic composition” was determined from ICP‐OES and gathered in Table S4.

The mechanism of glycerol oxidation involving several steps, starts with the adsorption of glycerol molecules, breaking of interatomic bonds, transfer of electrons, oxidation of the intermediates by oxygen species, and final desorption of the reaction products.^[28]^ Figure 5a–b displays the distribution of products obtained after anodic glycerol oxidation in CA and CP tests (Figures S15–S16 reports the evolution of the potential and current over time). The identified reaction products by HPLC are shown in Figure 5c–d in terms of the selectivity considering the experimental composition of Au and Pt metals; all quantitative metrics are reported in Tables S8 and S9. In glycerol oxidation, the cleavage of carbon‐carbon bonds can proceed via various pathways, yielding diverse products.[^25^, ^28^, ^81^] Bimetallic catalysts, such as those in the Au−Pt series, can influence this oxidation differently depending on their composition and the applied electrochemical method.[^81^, ^82^] The bar graphs indicate that the selectivity of anodic products in glycerol oxidation differs between CP and CA methods. Under CP conditions, the cleavage of C−C bonds tends to favor the formation of smaller molecules like formic acid, especially with higher platinum content. Conversely, increased gold content leads to a preference for glycolic acid, suggesting that the bond‐breaking mechanism leans more towards two‐carbon fragment products when gold′s catalytic effect is predominant. In CA tests, the data reveal that a theoretical balanced Pt and Au ratio of 1 : 1 (GDE‐Au_50_Pt_50_) optimizes glycolic acid selectivity. This finding suggests an ideal synergy between the two metals, enhancing carbon‐carbon bond cleavage towards a specific oxidation pathway with higher electron exchange, favoring glycolic acid production.^[73]^ Intriguingly, an increase in gold content slightly alters this selectivity, likely due to modifications in the catalyst′s electronic structure influencing the bond‐breaking mechanism. The variances in glycerol oxidation pathways under CP and CA conditions can be ascribed to differences in local electric fields and mass transport phenomena. During CA, fixing the potential may create conditions conducive to forming certain intermediates, leading to smaller organic molecules. In contrast, CP maintains a consistent current, potentially ensuring a more stable electron supply for the oxidation process, which could affect the stability of intermediate species differently and thereby influence product distribution. These results underscore the significance of methodological choices in glycerol electro‐oxidation.

The proposed reaction pathways are shown in Scheme S1. The selectivity of glycerol oxidation is significantly influenced by the metal components in the electrocatalysts. Gold exhibits high selectivity for primary oxidation products such TA, facilitating oxidative dehydrogenation of glycerol. Catalysts with higher Au content produce more primary products, indicating selective oxidation at the glycerol terminal positions (Figure 5). Conversely, platinum promotes further oxidation to secondary products like FA, and OA, enhancing overall oxidation. Catalysts with higher Pt content show increased production of these secondary products, suggesting deeper oxidation pathways. The combination of Au and Pt in bimetallic catalysts provides a synergistic effect, balancing the formation of primary and secondary products and improving overall selectivity. Our findings align with those reported in previous studies, where similar bimetallic catalysts have demonstrated varying selectivity depending on the composition and experimental conditions (see Supplementary Information, Table S6 for a detailed comparison). This comparative analysis highlights the influence of bimetallic composition and electrochemical methods on the product distribution of glycerol oxidation. In resume, analyzing glycerol oxidation products on GDE‐Au_100–x_Pt_x_ highlights the distinct selectivity between potentiostatic and galvanostatic conditions, revealing key insights into the influence of electrochemical methods on reaction outcomes. Additionally, by carefully selecting the electrolysis method and tuning the bimetallic composition, it is possible to manipulate the reaction pathways of glycerol oxidation, controlling the selectivity towards desired products. Understanding these nuances is essential for optimizing industrial electrolysis processes to improve yield and selectivity for targeted chemical production.

The post‐mortem analysis of the GDE‐Au_100–x_Pt_x_ electrodes is essential to understanding the stability of the electrocatalysts changes after glycerol oxidation. Here, we combined advanced characterization techniques, including SEM, EDX mapping, XRD, and ICP‐OES. Notably, the post‐mortem SEM‐EDX mapping analysis (as depicted in Figure 4e–f) reveals no significant morphological changes or physical transformations in the electrodes following the electrolysis process. This observation suggests that the physical structure of the electrodes remains robust, with no substantial morphological degradation or nanoparticle coalescence evident post‐electrolysis. Complementing the SEM, EDX spectra and quantitative data (reported in Figures S17–S18 and Table S10) offered a detailed elemental distribution across the electrode surface. Notably, EDX‐mapping identified a reduction in platinum content on the surface of the GDE‐Au_0_Pt_100_ electrode post‐electrolysis, indicating some loss of material during operation. Remarkably, the results indicated the overall stability of the bimetallic composition across the electrode surfaces, with no significant compositional changes detected for the alloys. XRD analysis was employed to scrutinize the crystallographic structure of the electrodes (Figure S19). The patterns acquired post‐electrolysis showed consistent peak positions when compared to pre‐electrolysis patterns, signifying that the crystalline structure and phase composition of the bimetallic particles remained unaltered. ICP‐OES measurement provided a quantitative confirmation of the surface observation. For the GDE‐Au_50_Pt_50_ sample, the atomic ratio of Au/Pt shifted from approximately 64 : 36 before electrolysis to 66 : 34 after electrolysis, supporting the EDX findings of platinum loss. This subtle alteration in the atomic ratio underscores a slight preferential dissolution or leaching of platinum from the catalytic surface during operation. The post‐mortem findings suggest that while the GDE‐Au_50_Pt_50_ electrode exhibits considerable stability, there is a nuanced balance between the two metals’ behavior during glycerol oxidation. Overall, all bimetallic electrodes, particularly the GDE‐Au_50_Pt_50_ electrode, demonstrate a promising level of durability, current density (during HER and GOR) and selectivity (GOR). These characteristic are crucial for the implement in hydroxide AEM‐based electrolysis systems, with the minor changes in metal composition offering insights for enhancing long‐term electrocatalytic performance.

### Performance in a Hydroxide AEM Based Electrolyzer

**Figure 6:**
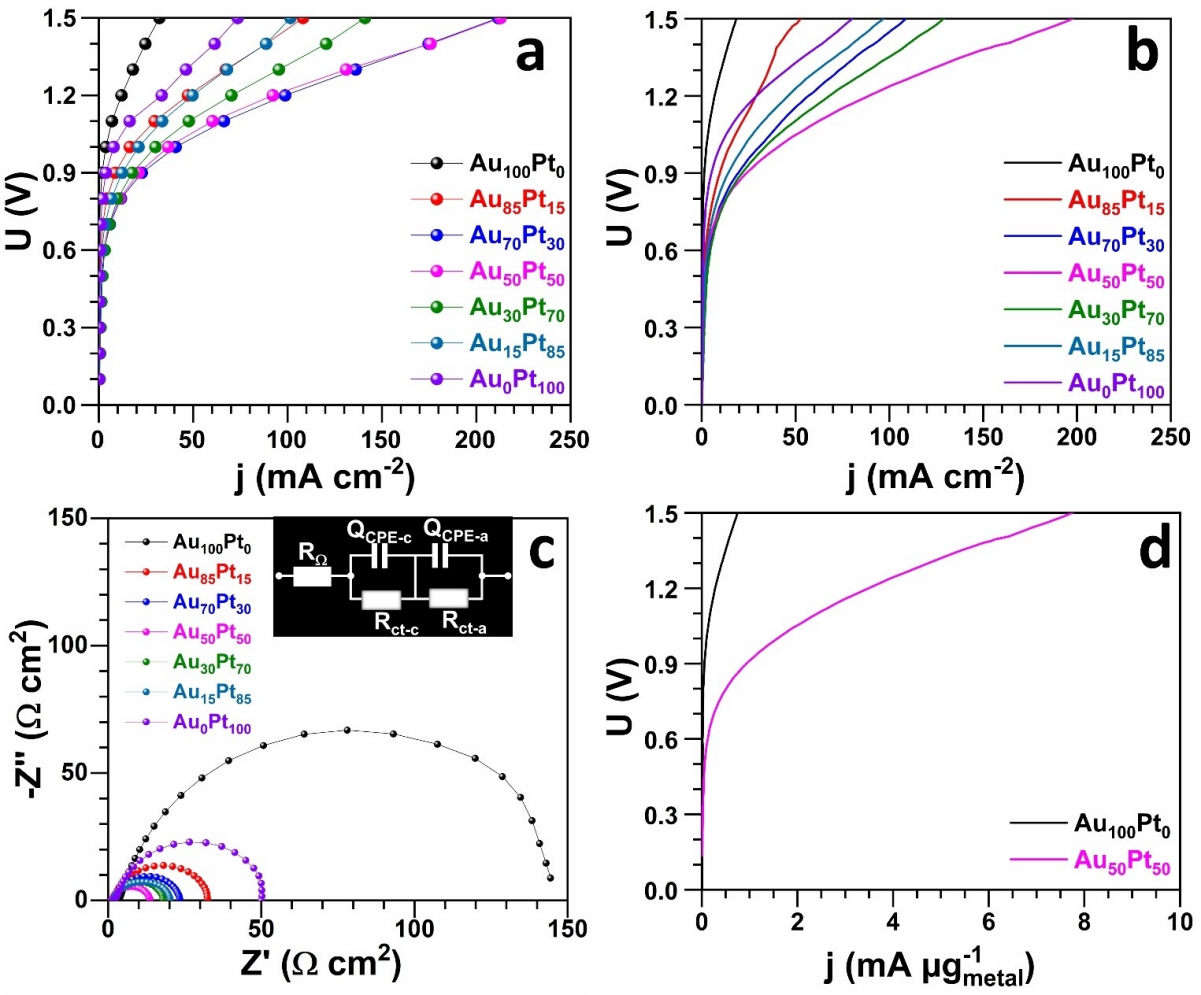
Performance of glycerol‐fed electrolyzer (GDE‐Au_100–x_Pt_x_‖GDE‐Au_100–x_Pt_x_) (50 °C). (a, b) Ohmic‐drop uncorrected polarization curves by: a) galvanostatic method (step of 0.05 V), and b) potentiostatic method (0.05 V s^−1^ scan rate). c) Nyquist impedance plots at 0.8 V. d) Mass‐dependent cell voltage (most active in current generation). Catholyte: 1 M NaOH (45 mL min^−1^, 50 °C). Anolyte: 1 M NaOH+0.1 M glycerol (23 mL min^−1^, 50 °C). Hydroxide anion exchange membrane: Sustainion® X37‐50 grade RT (5 cm^2^). Note: GDE‐Au_X_Pt_y_ refers to as theoretical composition and the true experimental “bulk atomic composition” was determined from ICP‐OES and gathered in Table S4.

**Figure 7:**
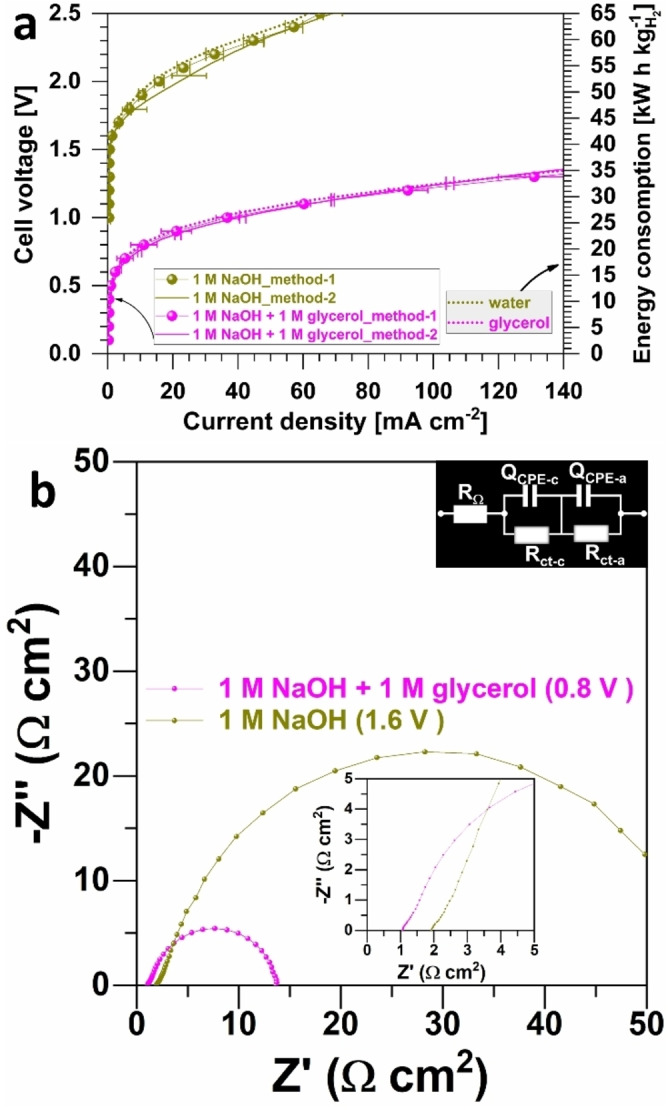
Electrolyzer performance (50 °C): effect of the presence of glycerol. a) Polarization curves by galvanostatic method (step of 0.05 V, method‐1) and potentiostatic method (0.05 V s^−1^ scan rate, method‐2). Left y‐axis is the cell voltage (ohmic‐drop uncorrected) and right y‐axis is the energy consumption per kg of produced H_2_. b) Complex‐plane Nyquist impedance plots recorded at different cell voltages in the absence and presence of glycerol: inset is the EEC of *R*_Ω_+*Q*_CPE‐a_//*R*_ct‐a_+*Q*_CPE‐c_//*R*_ct‐c_. Catholyte: 1 M NaOH (45 mL min^−1^, 50 °C) and GDE‐Au_50_Pt_50_ (25 μg_Pt+Au_ cm^−2^). Anolyte: 1 M NaOH+x M glycerol (x=0 or 1, 23 mL min^−1^, 50 °C) and GDE‐Au_50_Pt_50_ (25 μg_Pt+Au_ cm^−2^). Hydroxide anion exchange membrane: Sustainion® X37‐50 grade RT (5 cm^2^). Error bars represent one standard deviation (*n*=3).

Having demonstrated that galvanostatic electroshock approach can be implemented to synthesis of low loading Au−Pt nanoalloys onto GDE, we finally evaluated the electrocatalysts in a practical framework using a zero‐gap hydroxide AEM‐based electrolyzer. This setup employed multifunctional electrodes that functioned as both anode and cathode in two‐electrode tests (see Figures S2–S3). The straightforward preparation of multifunctional electrodes not only simplifies the electrolyzer setup but also markedly enhances system efficiency. The zero‐gap design plays a key role in minimizing ohmic losses by reducing the distance between the anode and cathode.^[41]^ Moreover, the incorporation of a flow setup considerably improves the mass transport.^[15]^ The experiments were performed at 50 °C and the ohmic‐drop uncorrected polarization curves by either the potentiostatic method (0.05 V step, Figures 6a and S19b) or LSV method (0.05 V s^−1^ scan rate, Figures 6b and S19a). Here, we have chosen not to privilege one method and thus allow the community to exploit our data. The ohmic‐drop corrected polarization curves are reported in Figure S20a–b. The control experiment without and with glycerol is depicted in Figure 7 when GDE‐Au_50_Pt_50_ functions as both anode and cathode electrocatalyst, which clearly highlights the energy saving when glycerol is added at the anolyte. Figure 7a shows that both method leads to nearly the performance in spite of the fact that the dynamic method is likely to lead to an overestimation of performance because it is much difficult to achieve a quasi‐stationary regime even with slow rates of 0.005–0.001 V s^−1^. It is noteworthy that the application of the potentiostatic or galvanostatic method for organic molecule electrooxidation can lead to the accelerated poisoning of active sites over prolonged periods at constant voltage or current levels. This phenomenon arises from the accumulation of intermediates and reaction products. Pulsed electrolysis as adopted in half‐cells[^8^, ^42^] could be a solution even if the H_2_ hourly flow rate would be impacted.

Polarization curve analyses of GDE‐Au_1–x_Pt_x_‖GDE‐Au_1–x_Pt_x_ indicated that bimetallic electrodes required a lower cell voltage to achieve the same current density compared to pure GDE−Pt or GDE−Au electrodes, as depicted in Figure 6a–b (also see Figures S20–S21). Notably, the electrolyzer generated an absolute current density of 0.21 A cm^−2^ at 1.5 V using the galvanostatic method for GDE‐Au_50_Pt_50_‖GDE‐Au_50_Pt_50_ (refer to Figure 6a). This current was roughly 3 times that generated by monometallic electrodes at the same voltage, a testament to the superior HER and GOR activities of GDE‐Au_50_Pt_50_. This enhancement was further confirmed by the LSV method (Figure 6b). The improved activity is primarily due to enhanced reaction rates, likely resulting from the synergistic and electronic effects of Au−Pt alloying as demonstrated by half‐cell study and microstructural analysis. The incorporation of Au atoms into Pt particles (and vice versa) significantly alters the electronic structure of these particles,^[31]^ as evidenced by XPS measurements for the GDE‐Au_50_Pt_50_ sample. EIS results (Figure 6c) further elucidated the electrocatalytic dynamics of our system, particularly in relation to our hypothesis concerning ohmic losses and charge transfer resistance. Taking metal loading into account, Figure 6d shows that the bimetallic electrocatalyst leads to the best electrolyzer performance.

For typical water electrolysis, the depressed semicircle at high frequency corresponding to low values of Z’ is associated with HER that has a much faster kinetics than OER.^[43]^ In Figure 7a–b, the only change is glycerol at the anode, so the depressed semicircle at low frequency corresponding to high values of Z’ can be rationally assigned to anodic part. Hence, the electrochemical phenomena can be described by the equivalent electrochemical circuit (EEC) of *R*_Ω_+*Q*_CPE‐a_//*R*_ct–a_+*Q*_CPE–c_//*R*_ct–c_ (“a” refers to as anode and “c” refers to as cathode). Based on this assignment, the data of Figure 6c reveal that the bimetallic electrocatalysts exhibited significantly lower charge transfer resistance compared to monometallic counterparts. This reduction in charge transfer resistance, validating our hypothesis and the half‐cell results (Figure 3) and the microstructure results (Figure 4). The enhanced electron transfer efficiency in the bimetallic systems, with GDE‐Au_50_Pt_50_ being the best comprise for the two method of characterization (Figure 6a vs Figure 6b), highlights the effectiveness of alloying in enhancing the electrocatalytic kinetics and maintaining a fair mass activity (Figure 6d). These findings, in tandem with the XPS results, not only reinforce the superior electrocatalytic performance of the bimetallic electrocatalysts but also confirm their role in minimizing overall energy input, thus bolstering the hypothesized advantages of such configurations in H_2_ generation technology. It is worth mentioning that although there is a drastic decrease (nearly two times) of the needed electrical energy (right ordinate in Figure 7a) to produce 1 kg H_2_ when the anode is supplied with glycerol, the current density remains too low if substantial H_2_ productivity is targeted, that is, *j*=1–2 A cm^−2^.^[44]^ Nevertheless, for applications where large quantities of H_2_ are not required and access to electricity is complicated, the anode can be supplied with glycerol. In fact, current performance can be greatly enhanced by using a KOH electrolyte (with lower viscosity than NaOH used herein as proof‐of‐concept) and hiking the operating temperature to 70–90 °C.

To finally validate our system′s efficiency in maintaining a cell voltage below 1 V while generating H_2_, we compared the H_2_ flow rate from the electrolyzer‘s cathodic outlet with theoretical value (Faraday law), as illustrated in Figures S22–S23 at when the current was fixed (100 mA, 20 mA cm^−2^ so that all electrodes could be compared). The outlet hydrogen flow rates were measured by the water displacement method. During testing, the electrolyzer consistently produced hydrogen at rates closely aligning with theoretical expectations. Operating at a constant current, the electrolyzer achieved a hydrogen yield of approximately 75 % of the theoretical maximum due to gas retention within the electrolyzer system as well as potential leakage. These elements highlight the necessity of continuous optimization in both electrolyzer design and operation. Despite these technical issues, the achieved efficiency underscores the effectiveness of both the electrocatalysts and the electrolyzer design in hydrogen production, offering insights into areas for future enhancements to further improve efficiency and practical viability.

## Conclusions

This study demonstrated the role of Au−Pt bimetallic electrocatalysts in augmenting hydrogen production through biomass‐based hydroxide anion‐exchange membrane electrolysis. Employing pulsed galvanostatic electrodeposition, we successfully developed a library of free‐standing GDE‐Au_100–x_Pt_x_ electrocatalysts as multifunctional electrode, which selectively electro‐oxidize glycerol (GOR) and facilitate hydrogen evolution (HER) in an alkaline environment. This method enables to precisely grow small amount of metallic particles directly onto GDE, streamlining the synthesis and improving efficiency by eliminating the need for catalytic ink and binders and reducing nanoparticle agglomeration during operation and facilitating the membrane‐electrode‐assembly (MEA). Owing to its nanoalloyed features, the as‐synthesized free‐standing GDE‐Au_64_Pt_36_ electrode materials with a lattice strain of 1.5 % demonstrated a drastic enhancement of the catalytic performance with an onset potential below 0.6 V vs RHE during half‐cell study of GOR. The zero‐gap hydroxide AEM‐based electrolyzer, optimized for minimal ohmic resistance, charge transfer resistance and improved mass transport, exhibited a hydrogen production efficiency with nearly two times lower electrical energy consumption (below 40 kW h per kg of H_2_) by leveraging the ability of Pt to catalyze HER and Au to catalyze GOR at high current density. The research also revealed the impact of electrode composition and the electrochemical methods on the selectivity of GORR. Post‐mortem analysis confirmed the stability and durability of the bimetallic compositions, underscoring their potential for green H_2_ production. In summary, the enhanced performance of the bimetallic Au−Pt electrocatalysts in a zero‐gap electrolyzer setup with a significantly reduced electrocatalyst loading of 12–26 μg cm^−2^ marks a significant advancement in sustainable hydrogen production technology. This work paves the way for further optimization and application of these systems in the field of renewable energy for the efficient and simultaneous electro‐synthesize of high value‐added fuels and chemicals, H_2_ at the cathode and organics at the anode.

## Conflict of Interests

The authors declare no conflict of interest.

1

## Supporting information

As a service to our authors and readers, this journal provides supporting information supplied by the authors. Such materials are peer reviewed and may be re‐organized for online delivery, but are not copy‐edited or typeset. Technical support issues arising from supporting information (other than missing files) should be addressed to the authors.

Supporting Information

## Data Availability

The data that support the findings of this study are available from the corresponding author upon reasonable request.

## References

[1] Y. Zuo , S. Bellani , M. Ferri , G. Saleh , D. V. Shinde , M. I. Zappia , R. Brescia , M. Prato , L. De Trizio , I. Infante , F. Bonaccorso , L. Manna , Nat. Commun. 2023, 14, 4680. 37542064 10.1038/s41467-023-40319-5 PMC10403570

[2] D. Tonelli , L. Rosa , P. Gabrielli , K. Caldeira , A. Parente , F. Contino , Nat. Commun. 2023, 14, 5532. 37684237 10.1038/s41467-023-41107-x PMC10491841

[3] C. V. M. Inocêncio , Y. Holade , C. Morais , K. B. Kokoh , T. W. Napporn , Electrochem. Sci. Adv. 2023, 3, e2100206.

[4] Y. Xia , H. Cheng , H. He , W. Wei , Commun Eng 2023, 2, 22.

[5] A. J. Bard , L. R. Faulkner , Electrochemical Methods: Fundamentals and Applications, 1st ed., John Wiley & Sons, Inc., USA, 1980.

[6] L. Zeng , Z. Zhao , F. Lv , Z. Xia , S.-Y. Lu , J. Li , K. Sun , K. Wang , Y. Sun , Q. Huang , Y. Chen , Q. Zhang , L. Gu , G. Lu , S. Guo , Nat. Commun. 2022, 13, 3822. 35780239 10.1038/s41467-022-31406-0 PMC9250493

[7] W. Luo , H. Tian , Q. Li , G. Meng , Z. Chang , C. Chen , R. Shen , X. Yu , L. Zhu , F. Kong , X. Cui , J. Shi , Adv. Funct. Mater. 2024, 34, 2306995.

[8] W. Xi , P. Yang , M. Jiang , X. Wang , H. Zhou , J. Duan , M. Ratova , D. Wu , Appl. Catal. B: Env. 2024, 341, 123291.

[9] Y. Holade , N. Tuleushova , S. Tingry , K. Servat , T. W. Napporn , H. Guesmi , D. Cornu , K. B. Kokoh , Catal. Sci. Technol. 2020, 10, 3071.

[10] L. Fan , Y. Ji , G. Wang , Z. Zhang , L. Yi , K. Chen , X. Liu , Z. Wen , J. Energy Chem. 2022, 72, 424.

[11] Y. Ma , K. Wu , T. Long , J. Yang , Adv. Energy Mater. 2023, 13, 2203455.

[12] G. Ma , X. Zhang , G. Zhou , X. Wang , Chem. Eng. J. 2021, 411, 128292.

[13] Q. Qian , X. He , Z. Li , Y. Chen , Y. Feng , M. Cheng , H. Zhang , W. Wang , C. Xiao , G. Zhang , Y. Xie , Adv. Mater. 2023, 35, 2300935. 10.1002/adma.202300935 36964932

[14] B. Guenot , M. Cretin , C. Lamy , Int. J. Hydrogen Energy 2017, 42, 28128.

[15] M. S. E. Houache , R. Safari , U. O. Nwabara , T. Rafaïdeen , G. A. Botton , P. J. A. Kenis , S. Baranton , C. Coutanceau , E. A. Baranova , ACS Appl. Energ. Mater. 2020, 3, 8725.

[16] S. Verma , S. Lu , P. J. A. Kenis , Nat. Energy 2019, 4, 466.

[17] W.-J. Liu , Z. Xu , D. Zhao , X.-Q. Pan , H.-C. Li , X. Hu , Z.-Y. Fan , W.-K. Wang , G.-H. Zhao , S. Jin , G. W. Huber , H.-Q. Yu , Nat. Commun. 2020, 11, 265. 31937783 10.1038/s41467-019-14157-3 PMC6959317

[18] M. Braun , C. S. Santana , A. C. Garcia , C. Andronescu , Curr. Opin. Green Sustain. Chem. 2023, 41, 100829.

[19] D. M. Morales , D. Jambrec , M. A. Kazakova , M. Braun , N. Sikdar , A. Koul , A. C. Brix , S. Seisel , C. Andronescu , W. Schuhmann , ACS Catal. 2022, 12, 982.

[20] J. R. Barbosa , C. H. Paranhos , O. C. Alves , N. R. Checca , J. P. Serna , A. L. Rossi , J. C. M. Silva , Electrochim. Acta 2020, 355, 136752.

[21] R. Boukil , N. Tuleushova , D. Cot , B. Rebiere , V. Bonniol , J. Cambedouzou , S. Tingry , D. Cornu , Y. Holade , J. Mater. Chem. A 2020, 8, 8848.

[22] M. Weber , P. Collot , H. El Gaddari , S. Tingry , M. Bechelany , Y. Holade , ChemElectroChem 2018, 5, 743.

[23] M. S. E. Houache , K. Hughes , R. Safari , G. A. Botton , E. A. Baranova , ACS Appl. Mater. Interfaces 2020, 12, 15095. 32159321 10.1021/acsami.9b22378

[24] Y. Zhu , Q. Qian , Y. Chen , X. He , X. Shi , W. Wang , Z. Li , Y. Feng , G. Zhang , F. Cheng , Adv. Funct. Mater. 2023, 33, 2300547.

[25] N. Tuleushova , Y. Holade , D. Cornu , S. Tingry , Electrochem. Sci. Adv. 2023, 3, e2100174.

[26] M. T. M. Koper , Chem. Sci. 2013, 4, 2710.

[27] J. Joo , T. Uchida , A. Cuesta , M. T. M. Koper , M. Osawa , J. Am. Chem. Soc. 2013, 135, 9991. 23808962 10.1021/ja403578s

[28] T. Li , D. A. Harrington , ChemSusChem 2021, 14, 1472. 33427408 10.1002/cssc.202002669

[29] X. Yu , E. C. Dos Santos , J. White , G. Salazar-Alvarez , L. G. M. Pettersson , A. Cornell , M. Johnsson , Small 2021, 17, 2104288. 10.1002/smll.202104288 34596974

[30] P. Lertthahan , S. Yongprapat , A. Therdthianwong , S. Therdthianwong , Int. J. Hydrogen Energy 2017, 42, 9202.

[31] E. Bus , J. A. Van Bokhoven , J. Phys. Chem. C 2007, 111, 9761.

[32] F. H. B. Lima , J. Zhang , M. H. Shao , K. Sasaki , M. B. Vukmirovic , E. A. Ticianelli , R. R. Adzic , J. Phys. Chem. C 2007, 111, 404.

[33] A. Habrioux , W. Vogel , M. Guinel , L. Guetaz , K. Servat , B. Kokoh , N. Alonso-Vante , Phys. Chem. Chem. Phys. 2009, 11, 3573. 19421563 10.1039/b820668f

[34] M. Ø Pedersen , S. Helveg , A. Ruban , I. Stensgaard , E. Lægsgaard , J. K. Nørskov , F. Besenbacher , Surf. Sci. 1999, 426, 395.

[35] P. Strasser , S. Koh , T. Anniyev , J. Greeley , K. More , C. Yu , Z. Liu , S. Kaya , D. Nordlund , H. Ogasawara , M. F. Toney , A. Nilsson , Nat. Chem. 2010, 2, 454. 20489713 10.1038/nchem.623

[36] X. Wang , W. Guo , Y. Fu , J. Mater. Chem. A 2021, 9, 663.

[37] D. Kumar , Prog. Mater. Sci. 2023, 136, 101106.

[38] D. Siegmund , S. Metz , V. Peinecke , T. E. Warner , C. Cremers , A. Grevé , T. Smolinka , D. Segets , U.-P. Apfel , JACS Au 2021, 1, 527. 34467315 10.1021/jacsau.1c00092 PMC8395688

[39] W. Wu , Z. Tang , K. Wang , Z. Liu , L. Li , S. Chen , Electrochim. Acta 2018, 260, 168. 10.1016/j.electacta.2017.12.065 PMC5881604 29622818

[40] Y. Holade , H. Guesmi , J.-S. Filhol , Q. Wang , T. Pham , J. Rabah , E. Maisonhaute , V. Bonniol , K. Servat , S. Tingry , D. Cornu , K. B. Kokoh , T. W. Napporn , S. D. Minteer , ACS Catal. 2022, 12, 12563.

[41] H. Jiang , Y. Sun , B. You , Acc. Chem. Res. 2023, 56, 1421. 37229761 10.1021/acs.accounts.3c00059

[42] L. Du , H. Xiong , H. Lu , L. M. Yang , R. Z. Liao , B. Y. Xia , B. You , Exploration 2022, 2, 20220024. 37324802 10.1002/EXP.20220024 PMC10190983

[43] M. Ghaemi , J. Power Sources 2002, 111, 248.

[44] T. W. Napporn , Y. Holade , B. Kokoh , S. Mitsushima , K. Mayer , B. Eichberger , V. Hacker , Fuel Cells and Hydrogen: From Fundamentals to Applied Research (Eds.: V. Hacker , S. Mitsushima ), Elsevier, 2018, 175.

[45] J. González-Cobos , S. Baranton , C. Coutanceau , ChemElectroChem 2016, 3, 1694.

[46] B. Guenot , M. Cretin , C. Lamy , J. Appl. Electrochem. 2015, 45, 973.

[47] M. Taguchi , N. Schwalb , Y. Rong , D. C. Vanegas , N. Garland , M. Tan , H. Yamaguchi , J. C. Claussen , E. S. McLamore , The Analyst 2016, 141, 3367. 27121177 10.1039/c6an00069j

[48] M. Hong , J. Zou , Z.-G. Chen , in Thermoelectricity and Advanced Thermoelectric Materials Elsevier, 2021, pp. 73.

[49] A. L. Yeang , Z. Li , S. Grunsfeld , G. R. McAndrews , Y. Cai , C. J. Barile , M. D. McGehee , Cell Reports Physical Science 2023, 4, 101660.

[50] Y. Holade , D. P. Hickey , S. D. Minteer , J. Mater. Chem. A 2016, 4, 17154.

[51] P. Morandi , N. Tuleushova , S. Tingry , J. Cambedouzou , S. D. Minteer , D. Cornu , Y. Holade , ACS Appl. Energ. Mater. 2020, 3, 7560.

[52] A. Sharma , S. Bhattacharya , S. Das , K. Das , Metall. Mater. Trans. A 2014, 45, 4610.

[53] N. D. Nikolić , G. Branković , V. M. Maksimović , M. G. Pavlović , K. I. Popov , J. Solid State Electrochem. 2010, 14, 331.

[54] Z. H. Kavousi , A. B. Abderrahmane , M. Ghorbanloo , S. Tingry , D. Cornu , M. Bechelany , Y. Holade , Electrochim. Acta 2024, 490, 144275.

[55] F. Ye , Z. Wang , C. Xu , M. Yuan , P. Liu , W. Yang , G. Liu , Renewable Energy 2020, 145, 514.

[56] F. Fouda-Onana , N. Guillet , A. M. AlMayouf , J. Power Sources 2014, 271, 401.

[57] A. R. Poerwoprajitno , L. Gloag , S. Cheong , J. J. Gooding , R. D. Tilley , Nanoscale 2019, 11, 18995. 31403640 10.1039/c9nr05802h

[58] R. N. Goldberg , L. G. Hepler , Chem. Rev. 1968, 68, 229.

[59] D. H. Evans , J. J. Lingane , Journal of Electroanalytical Chemistry (1959) 1963, 6, 1.

[60] W. Xu , D. Du , R. Lan , J. Humphreys , D. N. Miller , M. Walker , Z. Wu , J. T. S. Irvine , S. Tao , Appl. Catal. B: Env. 2018, 237, 1101.

[61] M. Escudero-Escribano , A. Verdaguer-Casadevall , P. Malacrida , U. Grønbjerg , B. P. Knudsen , A. K. Jepsen , J. Rossmeisl , I. E. L. Stephens , I. Chorkendorff , J. Am. Chem. Soc. 2012, 134, 16476. 22998588 10.1021/ja306348d

[62] G. F. Harrington , A. Cavallaro , D. W. McComb , S. J. Skinner , J. A. Kilner , Phys. Chem. Chem. Phys. 2017, 19, 14319. 28537623 10.1039/c7cp02017a

[63] J. Kang , W. Liu , D. Sarkar , D. Jena , K. Banerjee , Phys. Rev. X 2014, 4, 031005.

[64] K. Wen , W. Lv , W. He , J. Mater. Chem. A 2015, 3, 20031.

[65] G. Chang , Z. Cai , H. Jia , Z. Zhang , X. Liu , Z. Liu , R. Zhu , Y. He , Int. J. Hydrogen Energy 2018, 43, 12803.

[66] X. Weng , Y. Liu , K.-K. Wang , J.-J. Feng , J. Yuan , A.-J. Wang , Q.-Q. Xu , Int. J. Hydrogen Energy 2016, 41, 18193.

[67] Y. Holade , K. Servat , J. Rousseau , C. Canaff , S. Poulin , T. W. Napporn , K. B. Kokoh , J. Electrochem. Soc. 2015, 162, H929.

[68] A. Usher , D. C. McPhail , J. Brugger , Geochim. Cosmochim. Acta 2009, 73, 3359.

[69] Y. Yu , S. J. Lee , J. Theerthagiri , S. Fonseca , L. M. C. Pinto , G. Maia , M. Y. Choi , Chem. Eng. J. 2022, 435, 134790.

[70] C. C. Lima , M. V. F. Rodrigues , A. F. M. Neto , C. R. Zanata , C. T. G. V. M. T. Pires , L. S. Costa , J. Solla-Gullón , P. S. Fernández , Appl. Catal. B: Env. 2020, 279, 119369.

[71] H. Du , K. Wang , P. Tsiakaras , P. K. Shen , Appl. Catal. B: Env. 2019, 258, 117951.

[72] R. G. Da Silva , S. Aquino Neto , K. B. Kokoh , A. R. De Andrade , J. Power Sources 2017, 351, 174.

[73] Y. Zhou , Y. Shen , J. Xi , X. Luo , ACS Appl. Mater. Interfaces. 2019, 11, 28953. 31318191 10.1021/acsami.9b09431

[74] Y. Zhou , Y. Shen , J. Xi , Appl. Catal. B: Env. 2019, 245, 604.

[75] M. Zeng , Y. Li , J. Mater. Chem. A 2015, 3, 14942.

[76] J. Masa , C. Andronescu , W. Schuhmann , Angew. Chem. Int. Ed. 2020, 59, 15298. 10.1002/anie.202007672 PMC7496542 32608122

[77] J. D. Benck , T. R. Hellstern , J. Kibsgaard , P. Chakthranont , T. F. Jaramillo , ACS Catal. 2014, 4, 3957.

[78] J. Luo , J.-H. Im , M. T. Mayer , M. Schreier , M. K. Nazeeruddin , N.-G. Park , S. D. Tilley , H. J. Fan , M. Grätzel , Science 2014, 345, 1593. 25258076 10.1126/science.1258307

[79] M. Rafiee , M. N. Mayer , B. T. Punchihewa , M. R. Mumau , J. Org. Chem. 2021, 86, 15866. 34546751 10.1021/acs.joc.1c01391

[80] A. M. Bond , Comprehensive Coordination Chemistry II, Elsevier, 2003, 208.

[81] D. Kim , L. S. Oh , Y. C. Tan , H. Song , H. J. Kim , J. Oh , ACS Catal. 2021, 11, 14926.

[82] L. Huang , X. Yu , L. Huang , X. Zhang , L. Gu , Y. Cao , W. Li , J. Hu , X. Cao , Green Chem. 2022, 24, 9721.

[83] R. Phillips , A. Edwards , B. Rome , D. R. Jones , C. W. Dunnill , Int. J. Hydrogen Energy 2017, 42, 23986.

[84] K. B. Kokoh , J. M. Léger , B. Beden , H. Huser , C. Lamy , Electrochim. Acta 1992, 37, 1909.

[85] M. E. Orazem , B. Tribollet , Electrochemical Impedance Spectroscopy, 2 ed, John Wiley & Sons, Inc. : Hoboken, New Jersey, USA, 2017.

[86] A. Lasia , Electrochemical Impedance Spectroscopy and its Applications Springer-Verlag: New York, NY, USA, 2014.

[87] K. Elsøe , L. Grahl-Madsen , G. G. Scherer , J. Hjelm , M. B. Mogensen , J. Electrochem. Soc. 2017, 164, F1419.

[88] P. Lettenmeier , R. Wang , R. Abouatallah , S. Helmly , T. Morawietz , R. Hiesgen , S. Kolb , F. Burggraf , J. Kallo , A. S. Gago , K. A. Friedrich , Electrochim. Acta 2016, 210, 502.

[89] S. Sun , Z. Shao , H. Yu , G. Li , B. Yi , J. Power Sources 2014, 267, 515.

[90] C. Rozain , P. Millet , Electrochim. Acta 2014, 131, 160.

[91] M. Chatenet , B. G. Pollet , D. R. Dekel , F. Dionigi , J. Deseure , P. Millet , R. D. Braatz , M. Z. Bazant , M. Eikerling , I. Staffell , P. Balcombe , Y. Shao-Horn , H. Schäfer , Chem. Soc. Rev. 2022, 51, 4583. 35575644 10.1039/d0cs01079k PMC9332215

