# Supplementary material for: Galvanostatic Electroshock Synthesis of Low Loading Au−Pt Nanoalloys Onto Gas Diffusion Electrodes as Multifunctional Electrocatalysts for a Glycerol‐Fed Electrolyzer
Source: ChemSusChem. 2024 Sep 4;17(24):e202400996. doi: 10.1002/cssc.202400996 (PMC11660746; doi:10.1002/cssc.202400996)
Supplement: Supplementary file 1 — Supporting Information [file CSSC-17-e202400996-s001.pdf]

ChemSusChem

Supporting Information

Galvanostatic Electroshock Synthesis of Low Loading Au–Pt Nanoalloys Onto Gas Diffusion Electrodes as Multifunctional Electrocatalysts for a Glycerol-Fed Electrolyzer

Zahra Hagheh Kavousi, Layal Abdallah, Massomeh Ghorbanloo, Valerie Bonniol, Bertrand Rebiere, David Cornu, Mikhael Bechelany,* and Yaovi Holade*

Supplementary Information

Galvanostatic Electroshock Synthesis of Low Loading Au-Pt Nanoalloys onto Gas Diffusion Electrodes as Multifunctional Electrocatalysts for a Glycerol-Fed Electrolyzer

Zahra Hagheh Kavousi+,[a, b] Layal Abdallah+,[a] Massomeh Ghorbanloo,[b] Valerie Bonniol,[a] Bertrand Rebiere,[c] David Cornu,[a, d] Mikhael Bechelany*[a, e] and Yaovi Holade*[a, d]

a. Institut Européen des Membranes, IEM, UMR 5635, Univ Montpellier, ENSCM, CNRS, 34090 Montpellier, France.
b. Department of Chemistry, Faculty of Sciences, University of Zanjan, P.O. Box: 4537138791, Zanjan, Iran.
c. Institut Charles Gerhardt Montpellier, ICGM UMR 5253, Univ Montpellier, ENSCM, CNRS, 34090 Montpellier, France.
d. French Research Network on Hydrogen (FRH2), Research Federation No. 2044 CNRS, BP 32229, Nantes CEDEX 3 44322, France.
e. Functional Materials Group, Kuwait Gulf University for Science and Technology (GUST), Mubarak Al-Abdullah 32093, Kuwait.

*Corresponding authors.

E-mail addresses: mikhael.bechelany@umontpellier.fr (M.B.), yaovi.holade@enscm.fr (Y.H).

+These authors contributed equally.

TABLE OF CONTENTS

SUPPLEMENTARY TABLES..5

Table S1. Atomic Ratio Determined from EDX Analysis. 5

Table S2. Atomic Ratio Determined from EDX Analysis. 6

Table S3. Particle Size Determination of Catalysts from XRD Analysis. 7

Table S4. Quantitative Data from ICP-OES Analysis. 8

Table S5. Fitted EIS Data for HER in 1 M NaOH at 25 °C. 9

Table S6. Comparison of the performance of relevant metallic catalysts for the glycerol electrooxidation reaction in alkaline media from literature. 10

Table S7. Comparative performance of relevant metallic catalysts for HER in alkaline media from literature. 12

Table S8. Quantitative Electrolysis Data and HPLC Results Obtained via Chronopotentiometry. 13

Table S9. Quantitative Electrolysis Data and HPLC Results Obtained via Chronoamperometry. 14

Table S10. Post-Mortem Atomic Ratio Determined from EDX Analysis. 16

Table S11. The comparison of overall electrolysis performance coupling glycerol oxidation reactions with HER in aqueous media for electrocatalysts in recent reported. T: temperature. Empty box (–) means that the original article does not provide such data. 17

SUPPLEMENTARY FIGURES AND SCHEMES..18

Scheme S1. Reaction pathways for GOR in alkaline media.[26] 18

Figure S1. Steady-state CV recorded at 1 mV s^{-1} in H_2-saturated electrolyte (25 °C) for the calibration of the reference electrode: RHE vs MOE...19

Figure S2. Schematic of Zero-Gap AEM-Based Electrolyzer (5 cm²) and water displacement system. This figure provides a detailed schematic representation of a zero-gap anion exchange membrane-based electrolyzer with an active area of 5 cm², illustrating its design and component arrangement. 20

Figure S3. Zero-Gap AEM-Based Electrolyzer (5 cm²). a) Assembled configuration, and b) Disassembled components. 21

Figure S4. SEM-EDX mapping images across varied currents and durations: (a) -4.5 mA (60 min), (b) -4.5 mA (30 min), (c) -9 mA (15 min), (d) -9 mA (30 min), (e) -18 mA (7.5 min), and (f) -18 mA (15 min). 22

Figure S5. SEM images and related size distribution of the synthesized nanoparticles: (a1-a2) -4.5 mA (60 min), (b1-b2) -4.5 mA (30 min), (c1-c2) -9 mA (15 min), (d1-d2) -9 mA (30 min), (e1-e2) -18 mA (7.5 min), and (f1-f2) -18 mA (15 min). 23

Figure S6. Electrochemical performance of the GDE-$Au_{100-x}Pt_x$ (x = 50) across different durations and currents: a) CV measured at a scan rate of 100 mV s^{-1} in 1 M NaOH at 25 °C, b) Forward scan of the CV of glycerol (0.1 M) oxidation reaction (50 mV s^{-1}, 1 M NaOH, 25 °C), c) LSV curves of HER (5 mV s^{-1}, 1 M NaOH, 25 °C) and d) Nyquist impedance plots at -0.155 V vs RHE. Potentials are iR-drop uncorrected. 24

Figure S7. SEM images for electrodes with varying compositions: a) GDE-$Au_{100}Pt_0$, b) GDE-$Au_{85}Pt_{15}$, c) GDE-$Au_{70}Pt_{30}$, d) GDE-$Au_{30}Pt_{70}$, e) GDE-$Au_{15}Pt_{85}$, and f) GDE-Au_0 Pt_{100}.... 25

Figure S8. SEM-EDX mapping images of GDE-Au-Pt electrodes. This figure presents the atomic distribution for electrodes with varying compositions: a) GDE-$Au_{100}Pt_0$, b) GDE-$Au_{85}Pt_{15}$, c) GDE-$Au_{70}Pt_{30}$, d) GDE-$Au_{30}Pt_{70}$, e) GDE-$Au_{15}Pt_{85}$, and f) GDE-Au_0Pt_{100}..... 26

Figure S9. Representative survey XPS spectra of the as-synthesized GDE-$Au_{100-x}Pt_x$ (x = 0, 50, 100) electrodes. 27

Figure S10. Cyclic Voltammetry of GDE-$Au_{100-x}Pt_x$ electrodes. at 50 mV s^{-1} in 1 M NaOH at 25 °C.......... 28

Figure S11. HPLC Chromatograms of Standard Compounds (possible reaction products): (a) standard compounds ranging in concentration from 0.25 to 2 mM and (b) standard compounds prepared in different medium. 29

Figure S12. Calibration curves for expected standard reactants. 30

Figure. S13. Chromatogram obtained by HPLC of an electrolyte sample taken after GOR including an exemplifying product assignment. The example corresponds to a sample taken after 1 h electrolysis performed at 20 mA for CP and 0.89 V vs RHE for CA, in 1 M NaOH containing initially 100 mM glycerol.......... 31

Figure. S14. Diluted HPLC chromatogram of electrolyte after glycerol oxidation reaction (GOR) in H-type cell. This figure illustrates chromatograms of samples post 1-hour electrolysis at 20 mA for CP and 0.89 V vs RHE for CA, conducted in 1 M NaOH containing 1 M glycerol initially. The chromatograms highlight the product distribution and identification.......... 32

Figure. S15. CA curve during bulk electrolysis in a H-type cell at xx V vs RHE (1 M NaOH, 1 M glycerol, 25 °C, hydroxide AEM).......... 33

Figure. S16. CP curve during bulk electrolysis in a H-type cell at 20 mA cm^{-2} (1 M NaOH, 1 M glycerol, 25 °C, hydroxide AEM).......... 34

Figure S17. Post-Mortem SEM Images of GDE-Au-Pt Electrodes. This figure presents SEM images showing the morphology of the electrodes after use, with compositions as follows: (a) GDE-$Au_{100}Pt_0$, b) GDE-$Au_{85}Pt_{15}$, c) GDE-$Au_{70}Pt_{30}$, d) GDE-$Au_{50}Pt_{50}$, e) GDE-$Au_{30}Pt_{70}$, f) GDE-$Au_{15}Pt_{85}$, and g) GDE-Au_0Pt_{100}. 35

Figure S18. Post-Mortem SEM-EDX Mapping of GDE-Au-Pt Electrodes. Each panel shows the elemental distribution for different metal compositions: a) GDE-$Au_{100}Pt_0$, b) GDE-$Au_{85}Pt_{15}$, c) GDE-$Au_{70}Pt_{30}$, d) GDE-$Au_{30}Pt_{70}$, e) GDE-$Au_{15}Pt_{85}$, and f) GDE-Au_0 Pt_{100}.... 36

Figure S19. Post Mortem XRD Analysis of GDE-$Au_{100-x}Pt_x$ Electrodes (after 1 h glycerol electrooxidation (1 M glycerol) using CP methodology (j_{apply}= 20 mA cm^{-2}, 1 M NaOH, 50 °C). This figure illustrates the X-ray diffraction patterns of GDE-$Au_{100-x}Pt_x$ electrodes after usage, revealing their crystalline structure and compositional changes.......... 37

Figure S20. Polarization curves with error bars for GDE-$Au_{100-x}Pt_x$ ∥ GDE-$Au_{100-x}Pt_x$ Zero-gap glycerol electrolyzer: (a) the linear sweep voltammetry (LSV) strategy and (b) the staircase chronoamperometry strategy, highlighting performance under each method. Catholyte: 1 M NaOH (45 mL min^{-1}, 50 °C). Anolyte: 1 M NaOH + 1 M glycerol (23 mL min^{-1}, 50 °C). Hydroxide anion exchange membrane: Sustainion® X37-50 grade RT (5 cm^2). 38

Figure S21. Polarization Curves for GDE-$Au_{100-x}Pt_x$ ∥ GDE-$Au_{100-x}Pt_x$ Zero-gap Glycerol Electrolyzer, the cell voltage were iR-drop corrected: (a) LSV and (b) the staircase

chronoamperometry strategy. Catholyte: 1 M NaOH (45 mL min^{-1}, 50 °C). Anolyte: 1 M NaOH + 1 M glycerol (23 mL min^{-1}, 50 °C). Hydroxide anion exchange membrane: Sustainion® X37-50 grade RT (5 cm^2). 39

Figure S22. Evolution of cell voltage for GDE-$Au_{100-x}Pt_x$ ‖ GDE-$Au_{100-x}Pt_x$ (iR-drop uncorrected) at a current density of 20 mA cm^{-2} (0.1 A). 40

Figure S23. Comparison of hydrogen flow rate from electrolyzer cathodic outlet to theoretical production rates at a current density of 20 mA cm^{-2} (0.1 A). 41

References 42

SUPPLEMENTARY TABLES

Table S1. Atomic Ratio Determined from EDX Analysis.

Entry	Atomic Percentage				
	C	O	Pt	Au	Au_xPt_{100-x} x=
-4.5 mA (30 min)	99.2 ± 0.1	0.5 ± 0.1	0.1 ±	0.2	59.8 ± 3.3
-4.5 mA (60 min)	97.5 ±0.2	0.6 ±0.1	0.7 ±0.1	1.2 ± 0.1	61.5 ± 0.6
-9 mA (15 min)	99.3± 0.2	0.5 ± 0.1	0.1± 0.1	0.2± 0.1	69.3 ± 3.0
-9 mA (30 min)	99.2 ± 0.1	0.3± 0.1	0.2 ± 0.1	0.2 ± 0.1	56.9 ± 1.4
-18 mA (7.5 min)	98.5 ± 0.7	0.6 ± 0.2	0.3 ± 0.2	0.6 ± 0.5	69.7 ± 1.8
-18 mA (15 min)	99.3 ± 0.1	0.4 ± 0.2	0.1± 0.1	0.2± 0.1	74.5 ± 4.4

Note: The electrodes analyzed in this table were electrodeposited with an initial balanced ratio of metals, of Au/Pt = 50/50. Variations in the current and duration of the electrodeposition process were systematically employed to achieve the desired morphology and electrocatalytic activity. The optimal electrode condition, identified as -9 mA (30 min), is subsequently referred to as GDE-$Au_{50}Pt_{50}$ in this study.

Table S2. Atomic Ratio Determined from EDX Analysis.

Entry	Atomic Percentage				
	C	O	Pt	Au	Au_xPt_{100-x} x=
GDE-$Au_{100}Pt_0$	97.8 ± 0.1	1.1 ± 0.2	-	1.1 ± 0.1	100
GDE-$Au_{85}Pt_{15}$	97.9 ± 0.1	1.9 ± 0.1	0.1± 0.1	0.2± 0.1	91.8 ± 1.8
GDE-$Au_{70}Pt_{30}$	97.9 ± 0.2	2.0 ± 0.3	0.1± 0.1	0.1 ± 0.1	83.8 ± 5.9
GDE-$Au_{50}Pt_{50}$	99.2 ± 0.1	0.3± 0.1	0.2 ± 0.1	0.2 ± 0.1	56.9 ± 1.4
GDE-$Au_{30}Pt_{70}$	98.0 ± 0.1	1.5 ± 0.1	0.2± 0.1	0.3± 0.1	54.8 ± 0.8
GDE-$Au_{15}Pt_{85}$	97.9± 0.2	1.8± 0.1	0.2± 0.1	0.1± 0.1	38.9 ± 3.9
GDE-Au_0Pt_{100}	97.8 ± 0.1	2.1 ± 0.1	0.2± 0.1	-	100

SD value of 0.1 means that the actual value is much smaller

Table S3. Particle Size Determination of Catalysts from XRD Analysis.

**Au (at.%)**	**100**	**85**	**70**	**50**	**30**	**15**
**$2\theta_{(111)}$(°)**	38.39	38.50	38.63	38.98	39.16	39.15
**$d_{(111)}$(Å)**	2.34	2.34	2.33	2.31	2.30	2.30
**$L_{(v)(111)}$(nm)**	53	31	27	25	24	9
**$a_{(111)}$(Å)**	4.06	4.05	4.03	4.00	3.98	3.98
**Δa/a(%)**		0.27	0.60	1.47	1.90	1.88

Table S4. Quantitative Data from ICP-OES Analysis.

Bulk density of the GDE-based carbon paper was determined by weighting three different pieces of 5 cm × 5 cm (total exposed geometric area = 2×25 cm^2 = 50 cm^2, not taking into account the 3D structure of the electrode itself), the value was: 4.55 ± 0.02 g cm^{-2}.

Entry	ICP-OES					Calculations									
	Used (mg)	Metal (wt%)		%RSD		Metal (μg)		carbon (mg)	GDE(cm²)		Loading (μg cm^{-2}): single face			at%	
		Au	Pt	Au	Pt	Au	Pt		both faces	single face	Au	Pt	Au+Pt	Au	Pt
GDE-Au_0Pt_{100}	18.9	0.00	0.01	0	16.05	0	1.89	18.90	4.2	2.1	0.0	0.9	0.9	0	100
GDE-$Au_{15}Pt_{85}$	18.3	0.06	0.07	1.24	1.4	10.98	12.81	18.28	4.0	2.0	5.5	6.4	11.8	46	54
GDE-$Au_{30}Pt_{70}$	17.6	0.09	0.07	0.29	2.7	15.84	12.32	17.57	3.9	1.9	8.2	6.4	14.6	56	44
GDE-$Au_{50}Pt_{50}$	18.0	0.18	0.10	0.66	1.88	32.40	18.00	17.95	3.9	2.0	16.4	9.1	25.5	64	36
GDE-$Au_{50}Pt_{50}$ (Post-mortem)	33.1	0.12	0.06	0.82	1.55	39.72	19.86	33.04	7.3	3.6	10.9	5.5	16.4	66	34
GDE-$Au_{70}Pt_{30}$	17.0	0.23	0.06	0.54	2.41	39.10	10.20	16.95	3.7	1.9	21.0	5.5	26.4	79	21
GDE-$Au_{85}Pt_{15}$	18.1	0.23	0.02	0.29	5.53	41.63	3.62	18.05	4.0	2.0	21.0	1.8	22.8	92	8
GDE-$Au_{100}Pt_0$	17.6	0.27	0.00	0.53	0	47.52	0	17.55	3.9	1.9	24.6	0.0	24.6	100	0

Table S5. Fitted EIS Data for HER in 1 M NaOH at 25 °C.

For normalization, the geometric surface area was used (0.25 cm^2). The applied potential is iR-drop uncorrected.

Entry	$E_{applied}$ (mV vs RHE)	cell resistance $R_\Omega(\Omega\ cm^2)$	charge transfer resistance $R_{ct}(\Omega\ cm^2)$	$\eta_{@-10\ mA/cm^2}$(V)
GDE-$Au_{100}Pt_0$	-155	1	5429	0.69
GDE-$Au_{85}Pt_{15}$	-155	1	648	0.45
GDE-$Au_{70}Pt_{30}$	-155	1	504	0.45
GDE-$Au_{50}Pt_{50}$	-155	1	49	0.21
GDE-$Au_{30}Pt_{70}$	-155	1	125	0.37
GDE-$Au_{15}Pt_{85}$	-155	1	1036	0.40
GDE-Au_0Pt_{100}	-155	1	549	0.42

Table S6. Comparison of the performance of relevant metallic catalysts for the glycerol electrooxidation reaction in alkaline media from literature.

The metal loading is normalized to the geometric area. WE: working electrode. C: carbon black Vulcan. MWCNTs: multi-walled carbon nanotubes. *L*: metal loading on the electrode (total), per square centimeter of the electrode. Gly.: glycerol. *T*: temperature. RT: room temperature. E_{onset}: onset potential. j_P: peak current density and expressed in either amps per milligram of metal (A mg^{-1}) or milliamps per square centimeter of the electrode (mA cm^{-2}). ACF: activated carbon felt electrode. GC: glassy carbon. GDE: gas diffusion electrode. CP: carbon paper. Empty box (–) means that the original article does not provide the data.

Ref.	Electrode material			Conditions		Selectivity	Performance (scan rate: 50 mV s^{-1})		
	Nanocatalyst (metal loading)	WE (area)	L (µg cm^{-2})	Electrolyte + Gly	T (°C)		E_{onset} (V vs RHE)	j_P (A mg^{-1})	j_P (mA cm^{-2})
Herein	GDE-$Au_{100}Pt_0$	GDE (0.25 cm^{-2})	24.6	1 M NaOH + 0.1 Gly	25	For GDE-$Au_{50}Pt_{50}$, Glycerate and Glycolate	0.30	2.3	56.0
	GDE-$Au_{85}Pt_{15}$		22.8				0.24	2.6	59.7
	GDE-$Au_{70}Pt_{30}$		26.4				0.24	1.1	29.6
	GDE-$Au_{50}Pt_{50}$		25.5				0.23	2.5	61.9
	GDE-$Au_{30}Pt_{70}$		14.6				0.28	1.5	22.2
	GDE-$Au_{15}Pt_{85}$		11.8				0.27	4.4	51.61
	GDE-Au_0Pt_{100}		0.9				0.40	0.3	0.3
	Au/Vulcan (20 wt%, commercial)		6.2 (total amount of 12.4 µg)				0.75	0.4	2.2
	Pt/Vulcan (20 wt%, commercial)		0.3 (total amount of 0.6 µg)				0.62	0.9	0.2
2020[1]	AgPt/C	–	309.4 µg of Pt (electrode area was not indicated)	0.5 M NaOH + 1 M Gly	–	Not selective	0.25	1.8	–
2020[2]	GDE-AgAu(0.17 wt.%): 0.25 cm^2		16	1 M KOH + 0.1 Gly	25	Formate and Glycolate	0.3	18.2	290
	Au/C (20 wt.%)	CP (0.25 cm^2)	16	1 M KOH + 0.1 Gly	25	–	0.70	2.8	44
2019[3]	Pt-Co nanocubes	GC (0.196 cm^2)	10	1 M KOH + 0.1 Gly	30	–	0.5	1.2	*[10 μg_{Pt} cm^{-2}]*[1.2 mA $\mu g^{-1}{}_{Pt}$] = 12*
2019[4]	PtAg	GC (0.07 cm^2)	–	0.1 M KOH + 1 M Gly	–	DHA	0.50	–	7.57

2019[5]	Pt_4Au_6@Ag	GC (0.07 cm^2)	500	0.1 M KOH + 1 Gly	RT	DHA	0.50	–	3.1
2019[6]	Pt in N-doped graphene nanomesh on carbon cloth	GC (0.07 cm^2)	300	1 M KOH + 0.1 Gly	–	Formate	0.28	2.9	–
2018[7]	Pt_3Co_1 nanowires	GC (0.07 cm^2)	–	1 M KOH +1 M Gly	–	–	0.4	3.8	7.2
2017[8]	Ni@Pt/MWCNTs (40 wt%)	GC (0.07 cm^2)	320	1 M NaOH + 0.5 M Gly	–	Glycerate	0.49	0.27	79
2017[9]	Au_1Ag_1 (core/shell nanospheres)	GC (0.07 cm^2)	28.5	1 M KOH +1 M Gly	RT	–	0.97	3.5	–
2017[10]	$Pt_{86}Ru_{14}$/C (40 wt.%)	GC (0.07 cm^2)	320	1 M NaOH + 1 Gly	RT	DHA	0.5	0.6	–
2016[11]	$Pt_3Pd_6Bi_1$/C (40 wt.%)	GC (0.07 cm^2)	100	1 M NaOH + 0.1 Gly	RT	glyceraldehyde	0.4	–	40

Table S7. Comparative performance of relevant metallic catalysts for HER in alkaline media from literature.

The metal loading is normalized to the geometric area. *L*: metal loading on the electrode (total), per square centimeter of the electrode. *T*: temperature. RT: room temperature. Empty box (–) means that the original article does not provide the data.

Ref.	Electrode material			Conditions: T (°C)	Tafel Slope mV dec^{-1}	Overpotential at 10 mA cm^{-2} (mV vs RHE)
	Nanocatalyst (metal loading)	*L* (µg cm^{-2})	Electrolyte			
Herein	GDE-$Au_{100}Pt_0$	24.6	1 M NaOH	25	-	690
	GDE-$Au_{85}Pt_{15}$	22.8				450
	GDE-$Au_{70}Pt_{30}$	26.4				450
	GDE-$Au_{50}Pt_{50}$	25.5				210
	GDE-$Au_{30}Pt_{70}$	14.6				370
	GDE-$Au_{15}Pt_{85}$	11.8				400
	GDE-Au_0Pt_{100}	0.9				420
	Au/Vulcan (20 wt%, commercial)	6.2 (total amount of 12.4 µg)				620
	Pt/Vulcan (20 wt%, commercial)	0.3 (total amount of 0.6 µg)				560
2024[12]	Au@PtPd@Pt	-	1M KOH	RT	32	23
2019[13]	1.08 wt.% Pt/N-Mo_2C	210	1 M KOH	RT	108.63	101
2019[14]	Pt-Ni alloy	-	0.1 M KOH	25	-	82
2018[15]	$Au_{33}Pt_{67}$ NPs	-	0.1 M KOH	RT	73	88
2017[16]	Pt/Fe-NF	150	0.05 M KOH	RT	59.90	0.01
2016[17]	Pt-Ni/C	-	0.1M KOH	RT	59	70

Table S8. Quantitative Electrolysis Data and HPLC Results Obtained via Chronopotentiometry.

Entry	HPLC						Electrolysis		Efficiency	
	Name	ν_i	Concentration C_i(mM)	Selectivity (%)	z_i	Total charge of e^- (C)	Q_{OX} (C)	n_{exp}	FE_i(%)	FE (%)
GDE-Au_0Pt_{100}	OA	1.5	0.04	0.4	11	298.4	361.0	14.6	0.4	82.7
	TA	1.0	0.01	0.1	8				0.1	
	GeA	1.0	0.48	6.5	2				0.9	
	GoA	1.5	6.93	63.2	5				32.4	
	FA	3.0	6.53	29.8	8				48.8	
GDE-$Au_{15}Pt_{85}$	OA	1.5	0.10	0.4	11	355.8	361.0	6.5	1.0	98.6
	TA	1.0	0.64	3.9	8				4.8	
	GeA	1.0	6.08	36.9	2				11.4	
	GoA	1.5	13.28	53.7	5				62.1	
	FA	3.0	2.57	5.2	8				19.2	
GDE-$Au_{30}Pt_{70}$	OA	1.5	0.12	0.6	11	295.2	361.0	7.6	1.2	81.8
	TA	1.0	0.51	3.6	8				3.8	
	GeA	1.0	5.25	37.4	2				9.8	
	GoA	1.5	11.38	54.1	5				53.2	
	FA	3.0	1.83	4.4	8				13.7	
GDE-$Au_{50}Pt_{50}$	OA	1.5	0.12	0.5	11	292.4	361.9	7.4	1.2	80.8
	TA	1.0	0.52	3.6	8				3.9	
	GeA	1.0	5.88	40.5	2				11.0	
	GoA	1.5	11.22	51.6	5				52.4	
	FA	3.0	1.66	3.8	8				12.4	
GDE-$Au_{70}Pt_{30}$	OA	1.5	0.11	0.5	11	294.6	379.1	7.4	1.1	77.7
	TA	1.0	0.63	4.1	8				4.5	
	GeA	1.0	6.32	41.6	2				11.3	
	GoA	1.5	11.62	51.0	5				51.7	
	FA	3.0	1.29	2.8	8				9.2	
GDE-$Au_{85}Pt_{15}$	OA	1.5	0.10	0.4	11	289.7	363.2	7.1	1.0	79.8
	TA	1.0	0.79	5.2	8				5.9	
	GeA	1.0	6.36	41.8	2				11.8	
	GoA	1.5	11.46	50.2	5				53.3	
	FA	3.0	1.04	2.3	8				7.7	
GDE-$Au_{100}Pt_0$	OA	1.5	0.12	0.6	11	346.7	361.0	7.2	1.3	96.0
	TA	1.0	0.69	4.6	8				5.1	
	GeA	1.0	3.62	24.4	2				6.8	
	GoA	1.5	14.77	66.3	5				69.1	
	FA	3.0	1.84	4.1	8				13.8	

Table S9. Quantitative Electrolysis Data and HPLC Results Obtained via Chronoamperometry.

Entry	HPLC						Electrolysis		Efficiency	
	Name	ν_i	Concentration C_i(mM)	Selectivity (%)	z_i	Total charge of e^- (C)	Q_{OX} (C)	n_{exp}	FE_i(%)	FE (%)
GDE-Au_0Pt_{100}	OA	1.5	0.00	6.1	11	1.0	1.2	7.4	13.6	86.6
	TA	1.0	0.00	2.2	8				2.4	
	GeA	1.0	0.02	48.9	2				13.2	
	GoA	1.5	0.03	36.4	5				36.7	
	FA	3.0	0.01	6.4	8				20.7	
GDE-$Au_{15}Pt_{85}$	OA	1.5	0.05	2.2	11	31.8	38.3	7.3	5.0	83.0
	TA	1.0	0.01	0.8	8				0.9	
	GeA	1.0	0.63	41.0	2				11.2	
	GoA	1.5	1.21	52.1	5				53.2	
	FA	3.0	0.18	3.9	8				12.8	
GDE-$Au_{30}Pt_{70}$	OA	1.5	0.04	1.9	11	31.3	36.1	7.6	4.2	86.9
	TA	1.0	0.01	0.5	8				0.5	
	GeA	1.0	0.45	31.8	2				8.4	
	GoA	1.5	1.30	61.8	5				61.1	
	FA	3.0	0.17	4.0	8				12.7	
GDE-$Au_{50}Pt_{50}$	OA	1.5	0.04	1.1	11	56.2	69.3	8.2	2.2	81.1
	TA	1.0	0.06	2.2	8				2.2	
	GeA	1.0	0.65	25.8	2				6.3	
	GoA	1.5	2.57	68.2	5				62.5	
	FA	3.0	0.20	2.7	8				8.0	
GDE-$Au_{70}Pt_{30}$	OA	1.5	0.08	0.6	11	149.7	185.0	6.9	1.5	80.9
	TA	1.0	0.30	3.8	8				4.4	
	GeA	1.0	3.43	43.1	2				12.5	
	GoA	1.5	6.01	50.3	5				54.8	
	FA	3.0	0.52	2.2	8				7.6	
GDE-$Au_{85}Pt_{15}$	OA	1.5	0.07	0.5	11	170.8	203.3	6.6	1.3	84.0
	TA	1.0	0.33	3.6	8				4.4	
	GeA	1.0	3.76	41.5	2				12.5	
	GoA	1.5	7.15	52.6	5				59.4	
	FA	3.0	0.49	1.8	8				6.5	
GDE-$Au_{100}Pt_0$	OA	1.5	0.05	1.1	11	70.8	76.2	6.9	2.6	92.8
	TA	1.0	0.10	2.9	8				3.4	
	GeA	1.0	0.99	30.4	2				8.8	
	GoA	1.5	3.08	62.7	5				68.2	
	FA	3.0	0.28	2.8	8				9.9	

$$\nu = \frac{number\ of\ carbon\ atoms\ in\ the\ glycerol}{number\ of\ carbon\ atoms\ in\ the\ compound}$$

$$Selectivity = 100 \times \frac{{\nu_i}^{-1} Ci}{\sum_{i=1}^{i=5} {\nu_i}^{-1}\, Ci}$$

$$\Delta C = (C_0 - C_f) \approx \sum_{i=1}^{N} {\nu_I}^{-1} C_i$$

$$Q_{total} = \sum_{i=1}^{N} z_i C_i\, VF$$

$$FE = 100 \times \frac{VF}{Q_{ox}} \times \sum_{i=1}^{N} z_i C_i$$

where Q[C] is the charge, ΔC[mol L^{-1}] = the transformed concentration of glycerol, V[L] is the volume of the electrolysis solution, F[= 96485 C mol^{-1}] is the Faradaic constant, FE(%) is the faradaic yield or efficiency, n_{th} is the theoretical exchanged number of electrons, n_{exp} is the experimental exchanged number of electrons, z_i is the experimental exchanged number of electrons per molecule of glycerol, ν_i is the stoichiometric coefficient, and i is the compound.

Table S10. Post-Mortem Atomic Ratio Determined from EDX Analysis.

Entry	Atomic Percentage				
	C	O	Pt	Au	Au_xPt_{100-x} x=
GDE-$Au_{100}Pt_0$	97.8 ± 0.2	1.7 ± 0.2	0.0	0.5	100
GDE-$Au_{85}Pt_{15}$	97.9 ± 0.2	1.5 ± 0.2	0.0	0.5	95.4 ± 1.2
GDE-$Au_{70}Pt_{30}$	98.0 ± 0.3	1.4 ± 0.3	0.1	0.5	82.3 ± 0.2
GDE-$Au_{50}Pt_{50}$	98.1 ± 0.1	1.4 ± 0.1	0.2	0.4	69.5 ± 1.2
GDE-$Au_{30}Pt_{70}$	98.1	1.6	0.1	0.2	52.9 ± 6.6
GDE-$Au_{15}Pt_{85}$	98.0	1.7	0.1	0.1	43.2 ± 1.2
GDE-Au_0Pt_{100}	97.9 ± 0.2	2.0 ± 0.2	0.1	0	0

Table S11. The comparison of overall electrolysis performance coupling glycerol oxidation reactions with HER in aqueous media for electrocatalysts in recent reported. T: temperature. Empty box (–) means that the original article does not provide such data.

Cathode		Separator (temperature)	Anode			Product	Ref.
Electrocatalyst	Electrolyte		Electrocatalyst	Electrolyte	Cell voltage (V)		
GDE-$Au_{50}Pt_{50}$	1 M NaOH	Sustainion® X37-50 grade RT (50 °C)	GDE-$Au_{50}Pt_{50}$	1 M NaOH + 1M Glycerol	• 10 mA cm^{-2}: 0.75<br>• 50 mA cm^{-2}: 1.04<br>• 100 mA cm^{-2}: 1.23	Glycerate and Glycolate	This work
Pt/C catalyst	1 M KOH + 0.1 M Glycerol	-	$NiCo_2O_4$/NF	1 M KOH + 0.1 M Glycerol	• 10 mA cm^{-2}: 1.15<br>• 50 mA cm^{-2}: 1.34<br>• 100 mA cm^{-2}:1.45	Formate	2024[18]
Ir-Co_3O_4/NF	1 M KOH	Separator	Ir-Co_3O_4/NF	1.0 M KOH+ 0.1 M Glycerol	• 10 mA cm^{-2}: 1.40<br>• 50 mA cm^{-2}: 1.67<br>• 100 mA cm^{-2}: -	Formate	2023[19]
NiVRu-LDHs NAs/NF	1 M KOH	Nafion 115	NiVRuLDHs / NF	1 M KOH + 0.1 M Glycerol	• 10 mA cm^{-2}: 1.35<br>• 50 mA cm^{-2}: 1.50<br>• 100 mA cm^{-2}:1.62	Formate	2022[20]
NiCo hydroxide / CC	1 M KOH	Nafion 117	NiCo hydroxide / CC	1 M KOH + 0.1 M Glycerol	• 10 mA cm^{-2}: 1.33<br>• 50 mA cm^{-2}: 1.50<br>• 100 mA cm^{-2}:1.58	Formate	2022[21]
RhIr/Ti	0.5 M H_2SO_4	proton membrane (PEM)	CoNiCuMnMo-NPs/CC	1 M KOH + 0.1 M Glycerol	• 10 mA cm^{-2}: 0.55<br>• 50 mA cm^{-2}: 0.75<br>• 100 mA cm^{-2}: 0.90	Formate	2022[22]
MoO_x/Pt	1 M KOH	Nafion N-117 (25 °C)	MoO_x/Pt	1 M KOH + 0.1 M Glycerol	• 10 mA cm^{-2}: 0.70<br>• 20 mA cm^{-2}: 0.90	Glycerate	2021[23]
Pt/C/CP	5 mM H_2SO_4 + 0.2 M Glycerol	-	MnO_2/CP	5 mM H_2SO_4 + 0.2 M Glycerol	• 10 mA cm^{-2}: 1.38<br>• 20 mA cm^{-2}: 1.68	Formate	2021[24]
Ni-Mo-N/CFC	1 M KOH + 0.1 M Glycerol	-	Ni-Mo-N/CFC	1 M KOH + 0.1 M Glycerol	• 10 mA cm^{-2}: 1.36<br>• 50 mA cm^{-2}: 1.55<br>• 100 mA cm^{-2}: 1.82	Formate	2019[25]

SUPPLEMENTARY FIGURES AND SCHEMES

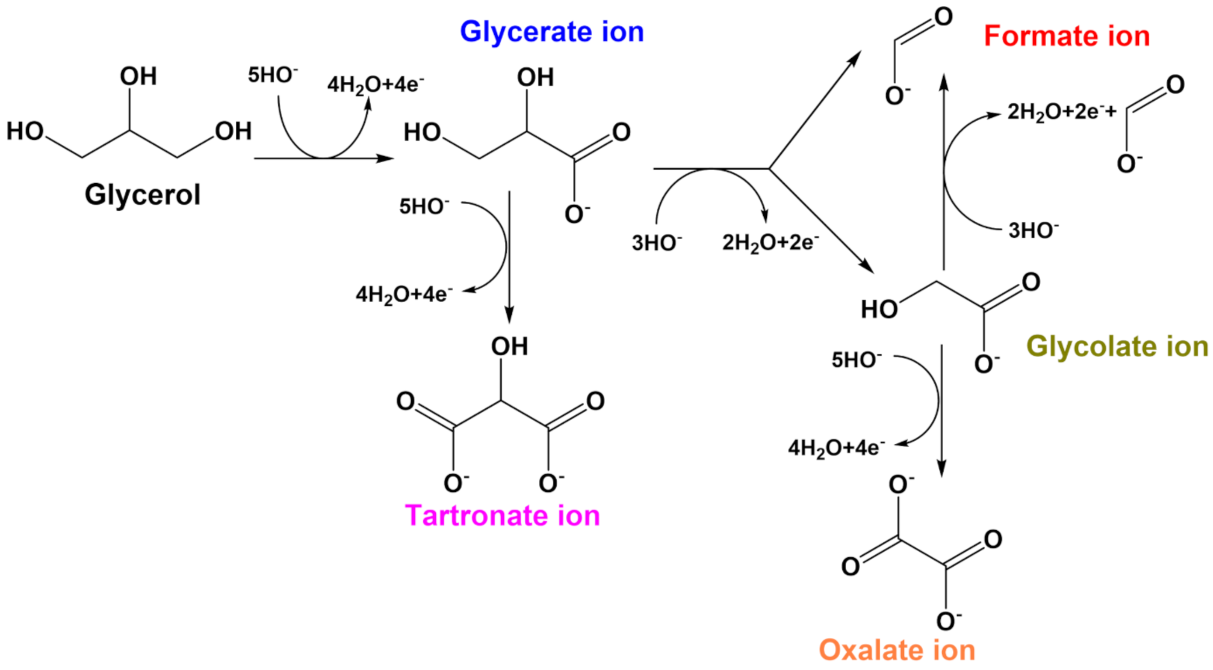

Scheme S1. Reaction pathways for GOR in alkaline media.[26]

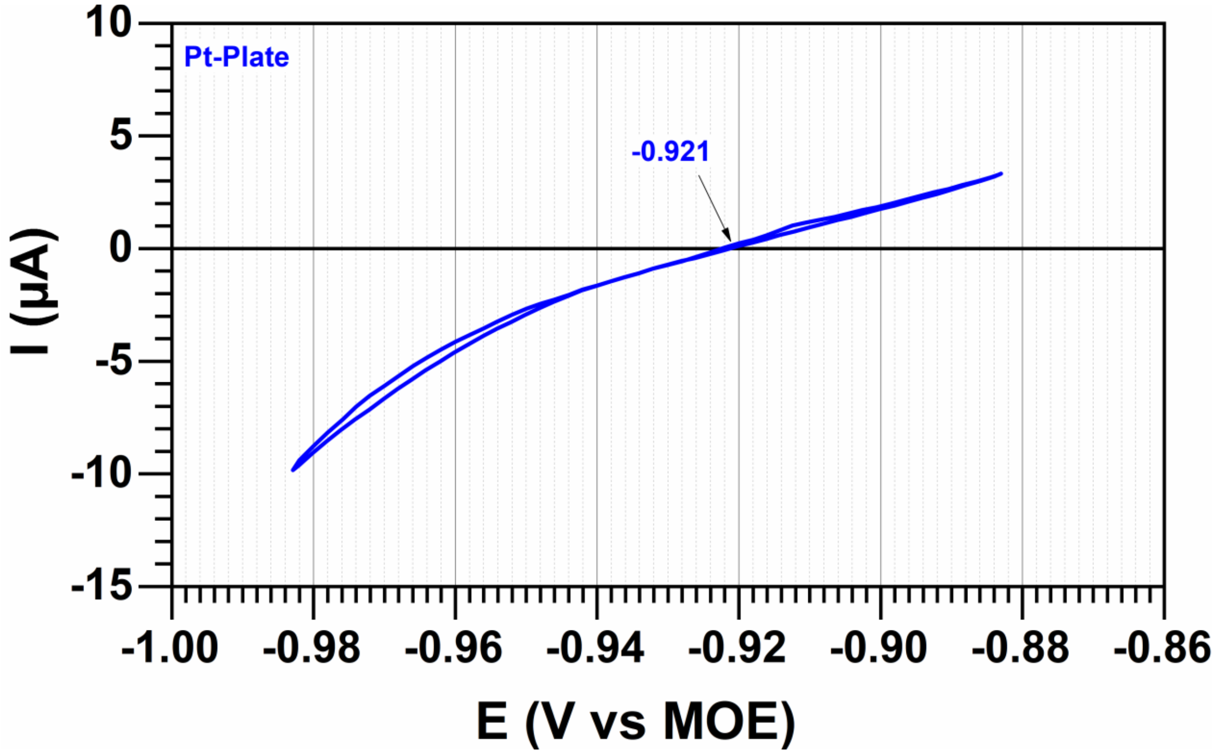

Figure S1. Steady-state CV recorded at 1 mV s^{-1} in H_2-saturated electrolyte (25 °C) for the calibration of the reference electrode: RHE vs MOE.

Figure S1 illustrates the calibration of the reference electrode through CV experiment, RHE with the MOE. This was achieved by a steady-state CV recorded in the electrolyte saturated with H_2. In this setup, a platinum plate served as the working electrode, while a glassy carbon electrode was used as the counter electrode. The reference electrode comprised a MOE electrode immersed in a 1 M MOH solution, referred to as MOE.

<u>Comment</u>: In our experimental setup, the thermodynamic potential for the RHE was determined by averaging the potentials at which the current reaches zero. For a concentration of 1 M NaOH, we employed the conversion formula:

E (V vs RHE) = E (V vs MOE) + 0.92.

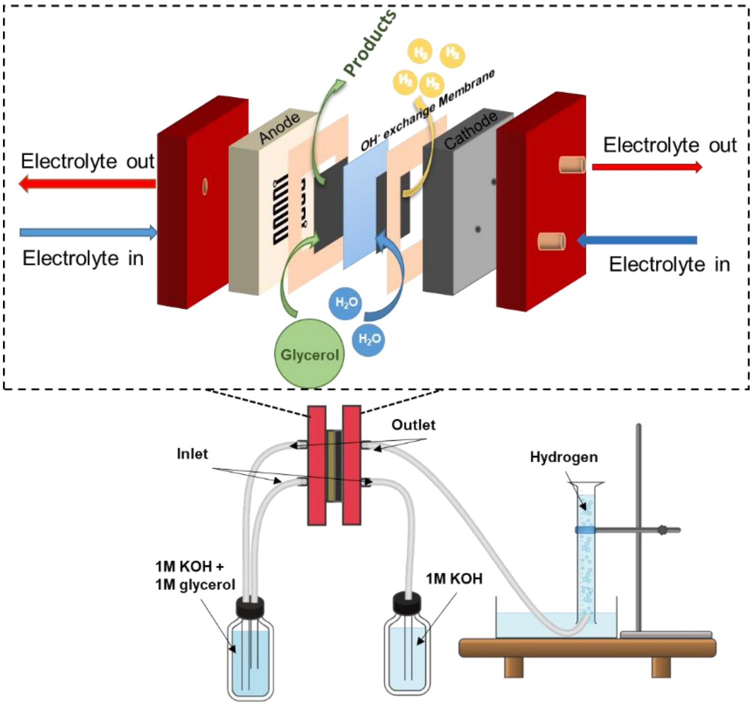

Figure S2. Schematic of Zero-Gap AEM-Based Electrolyzer (5 cm²) and water displacement system. This figure provides a detailed schematic representation of a zero-gap anion exchange membrane-based electrolyzer with an active area of 5 cm², illustrating its design and component arrangement as well as the water displacement method to measure H_2 volume.

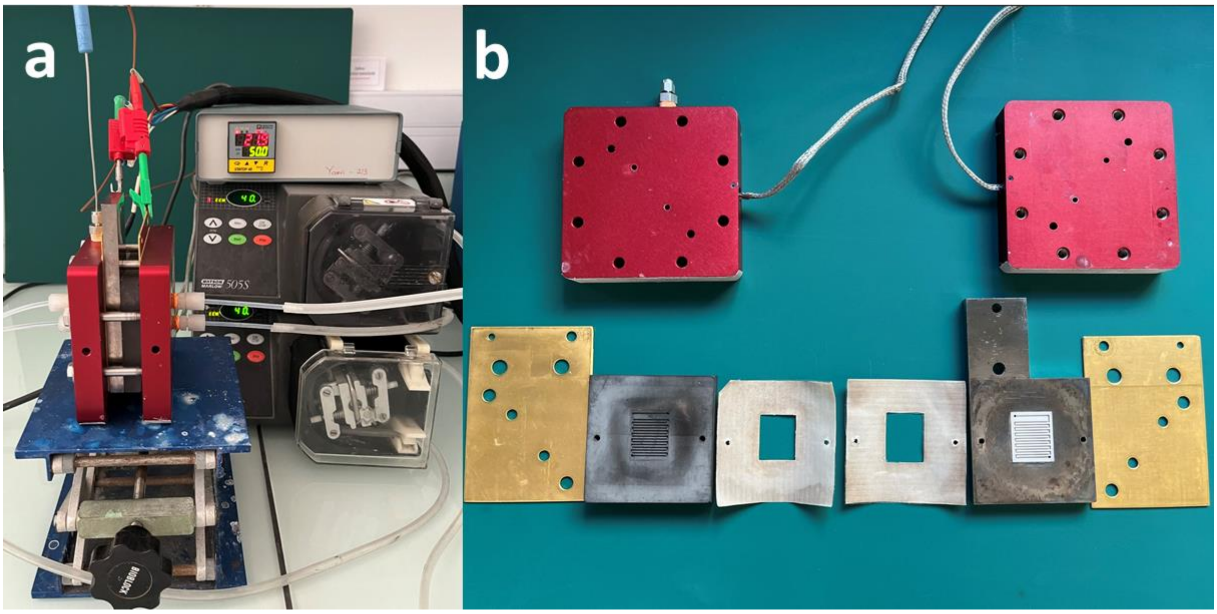

Figure S3. Zero-Gap AEM-Based Electrolyzer (5 cm^2). a) Assembled configuration, and b) Disassembled components.

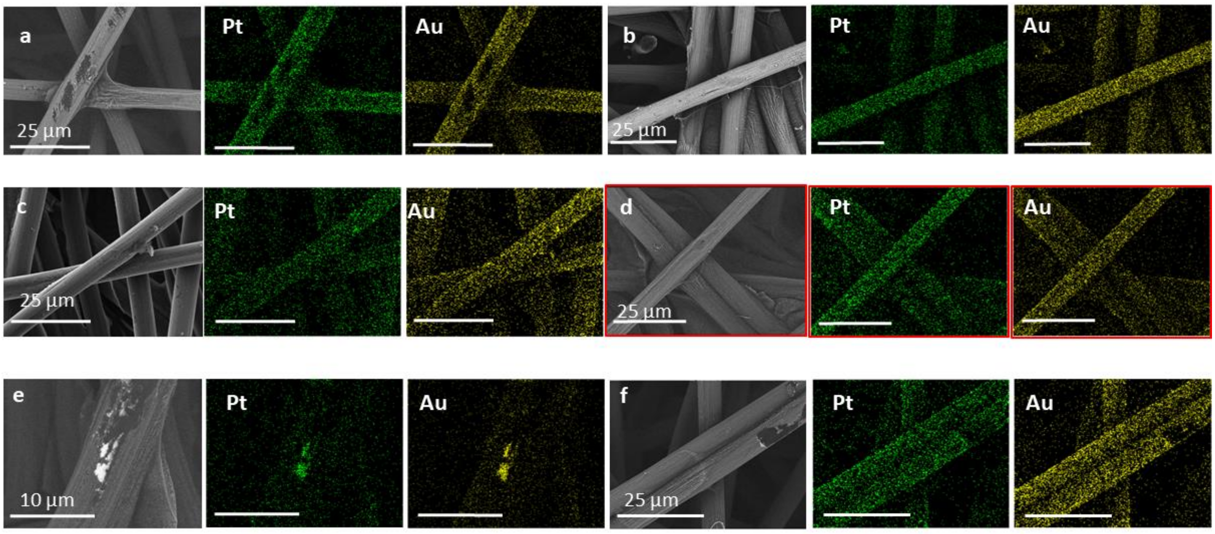

Figure S4. SEM-EDX mapping images across varied currents and durations: (a) -4.5 mA (60 min), (b) -4.5 mA (30 min), (c) -9 mA (15 min), (d) -9 mA (30 min), (e) -18 mA (7.5 min), and (f) -18 mA (15 min).

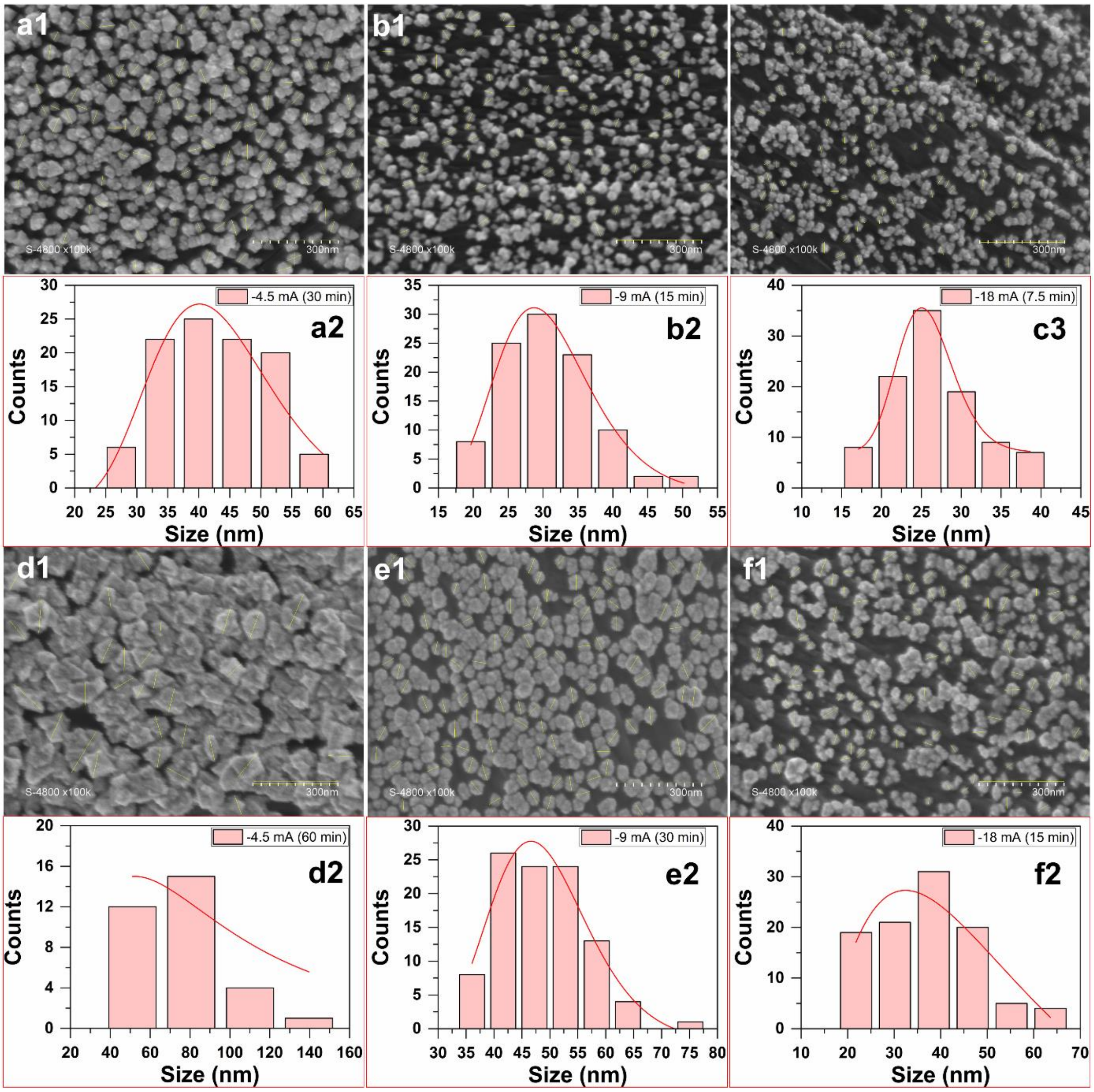

Figure S5. SEM images and related size distribution of the synthesized nanoparticles: (a1-a2) -4.5 mA (60 min), (b1-b2) -4.5 mA (30 min), (c1-c2) -9 mA (15 min), (d1-d2) -9 mA (30 min), (e1-e2) -18 mA (7.5 min), and (f1-f2) -18 mA (15 min).

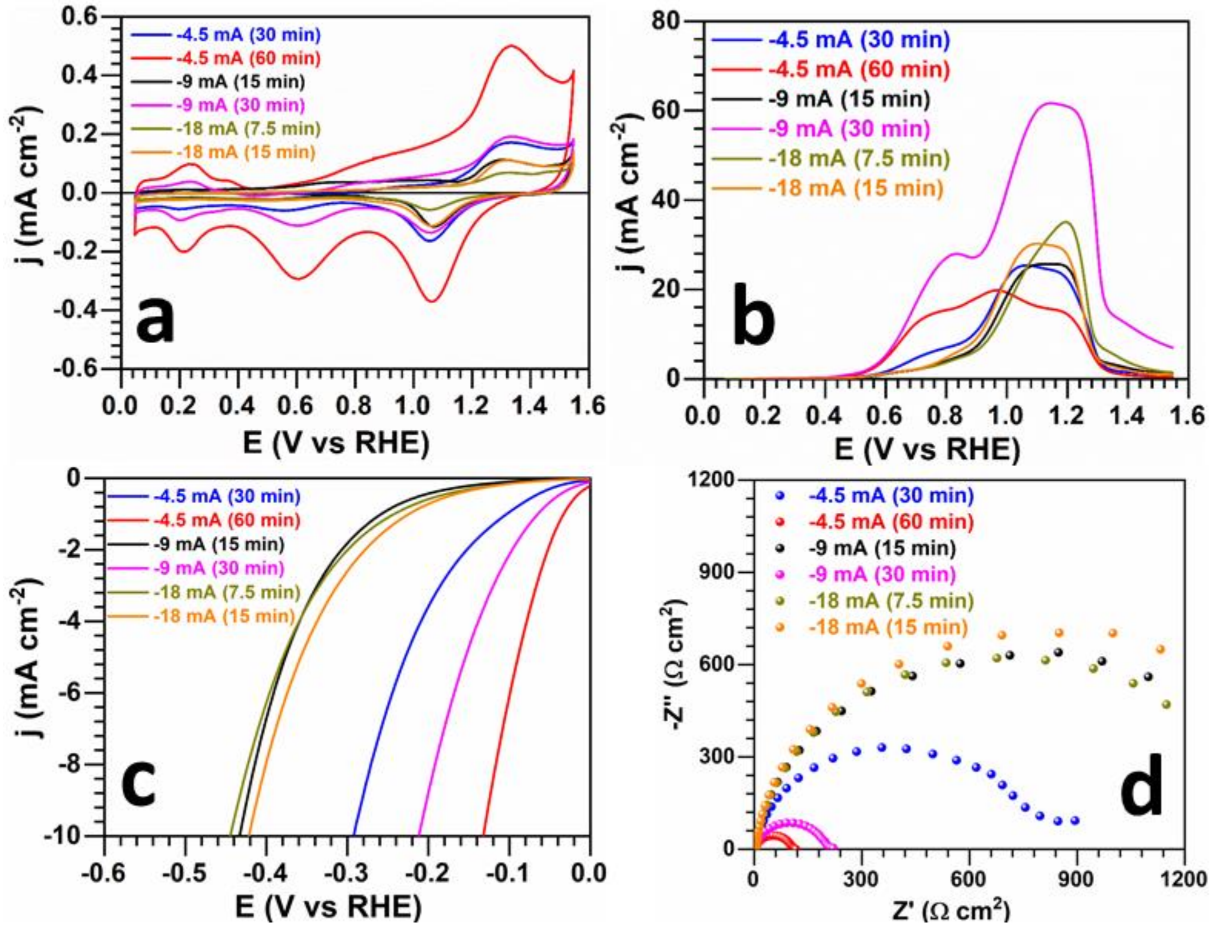

Figure S6. Electrochemical performance of the GDE-$Au_{100-x}Pt_x$ (x = 50) across different durations and currents: a) CV measured at a scan rate of 100 mV s^{-1} in 1 M NaOH at 25 °C, b) Forward scan of the CV of glycerol (0.1 M) oxidation reaction (50 mV s^{-1}, 1 M NaOH, 25 °C), c) LSV curves of HER (5 mV s^{-1}, 1 M NaOH, 25 °C) and d) Nyquist impedance plots at -0.155 V vs RHE. Potentials are iR-drop uncorrected.

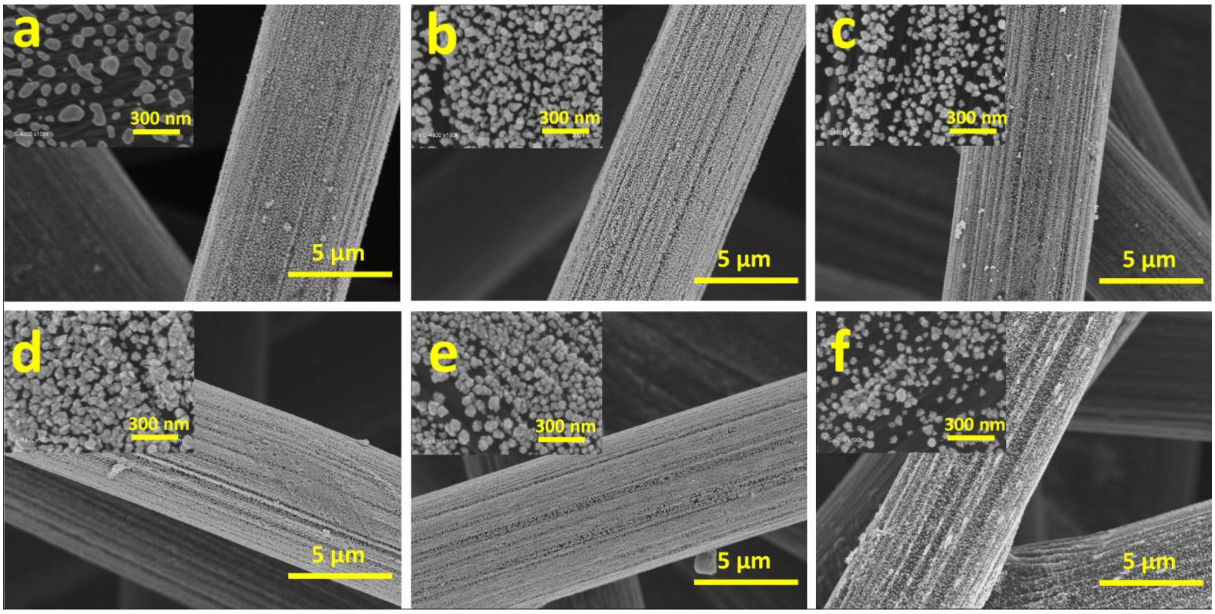

Figure S7. SEM images for electrodes with varying compositions: a) GDE-$Au_{100}Pt_0$, b) GDE-$Au_{85}Pt_{15}$, c) GDE-$Au_{70}Pt_{30}$, d) GDE-$Au_{30}Pt_{70}$, e) GDE-$Au_{15}Pt_{85}$, and f) GDE-Au_0 Pt_{100}.

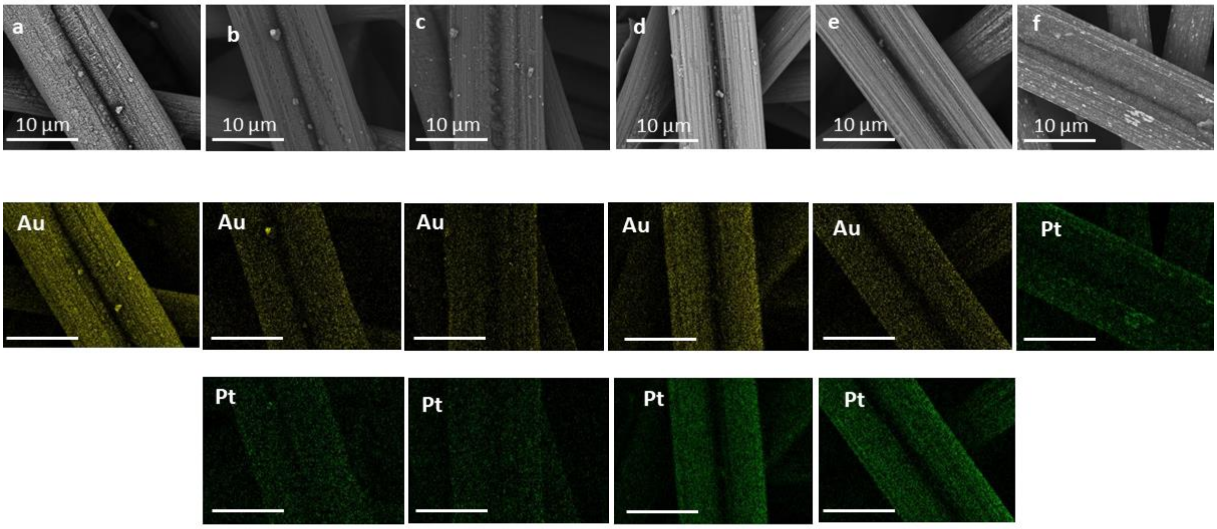

Figure S8. SEM-EDX mapping images of GDE-Au-Pt electrodes. This figure presents the atomic distribution for electrodes with varying compositions: a) GDE-$Au_{100}Pt_0$, b) GDE-$Au_{85}Pt_{15}$, c) GDE-$Au_{70}Pt_{30}$, d) GDE-$Au_{30}Pt_{70}$, e) GDE-$Au_{15}Pt_{85}$, and f) GDE-Au_0Pt_{100}.

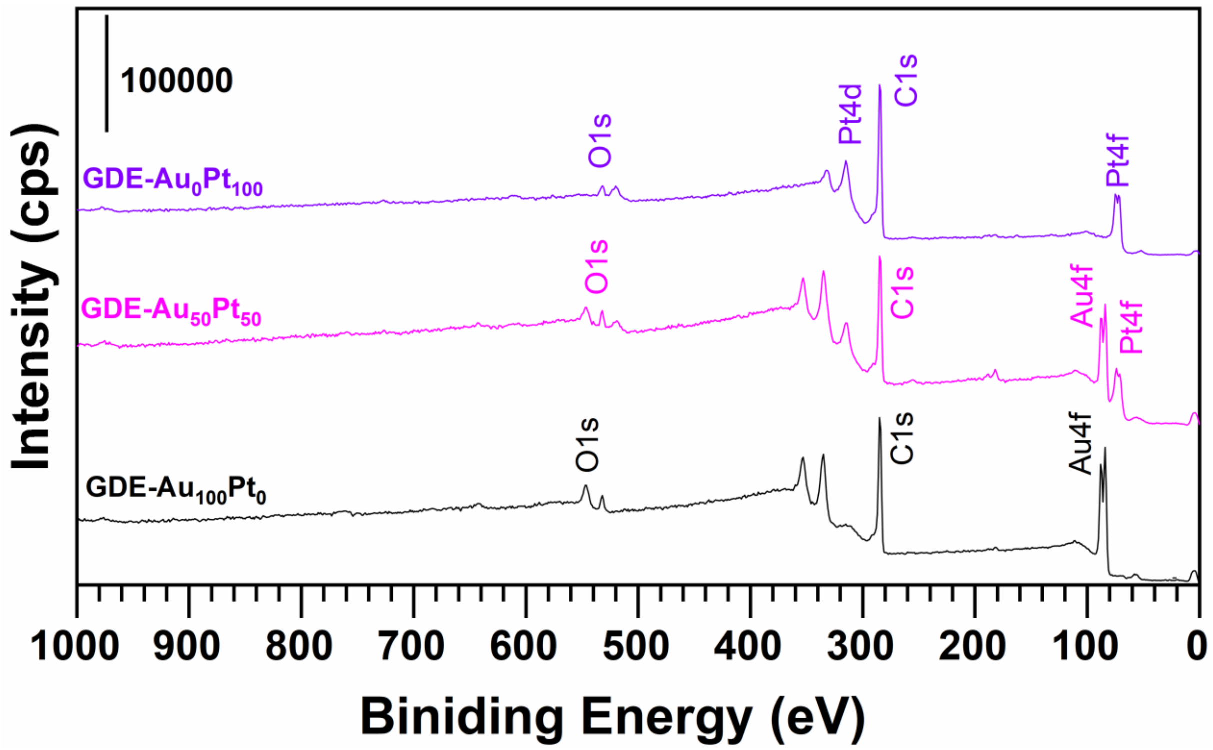

Figure S9. Representative survey XPS spectra of the as-synthesized GDE-$Au_{100-x}Pt_x$ (x = 0, 50, 100) electrodes.

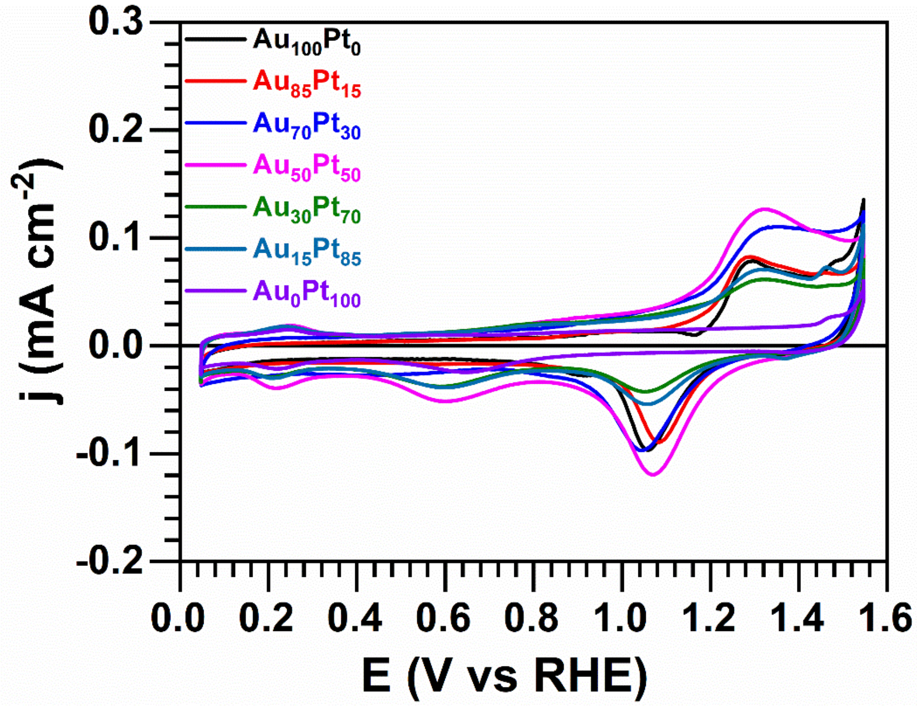

Figure S10. Cyclic Voltammetry of GDE-$Au_{100-x}Pt_x$ electrodes. at 50 mV s^{-1} in 1 M NaOH at 25 °C.

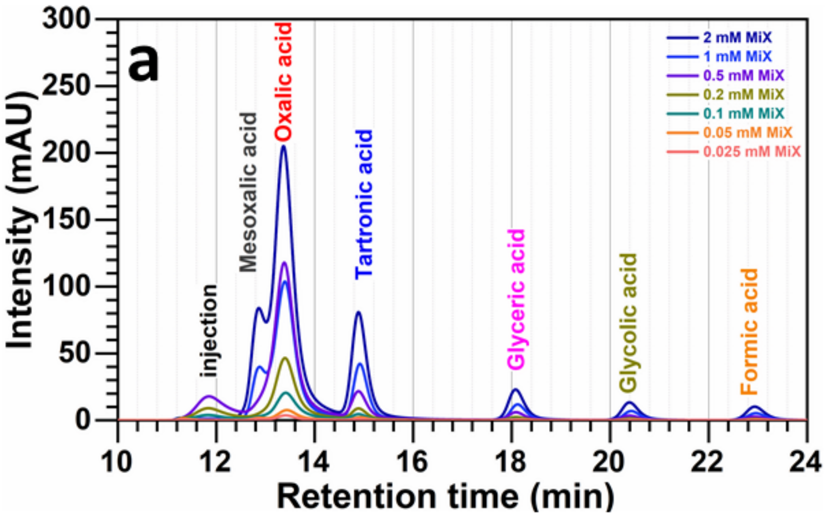

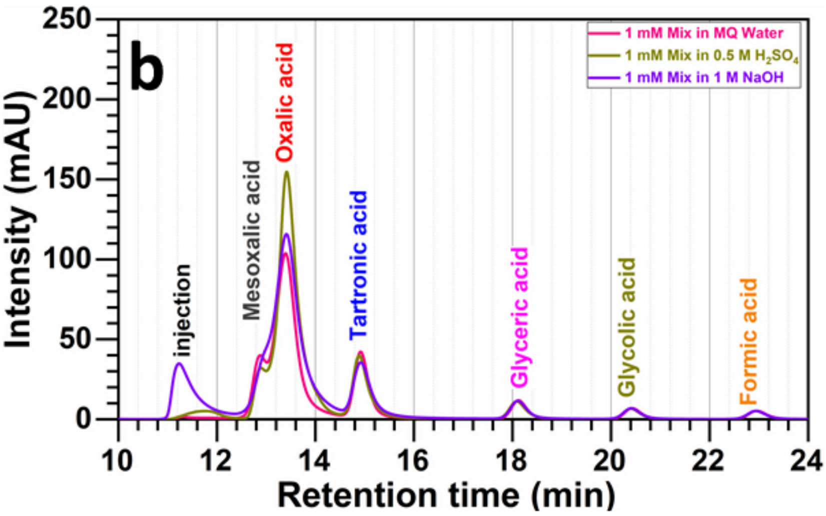

Figure S11. HPLC Chromatograms of Standard Compounds (possible reaction products): (a) standard compounds ranging in concentration from 0.25 to 2 mM and (b) standard compounds prepared in different medium.

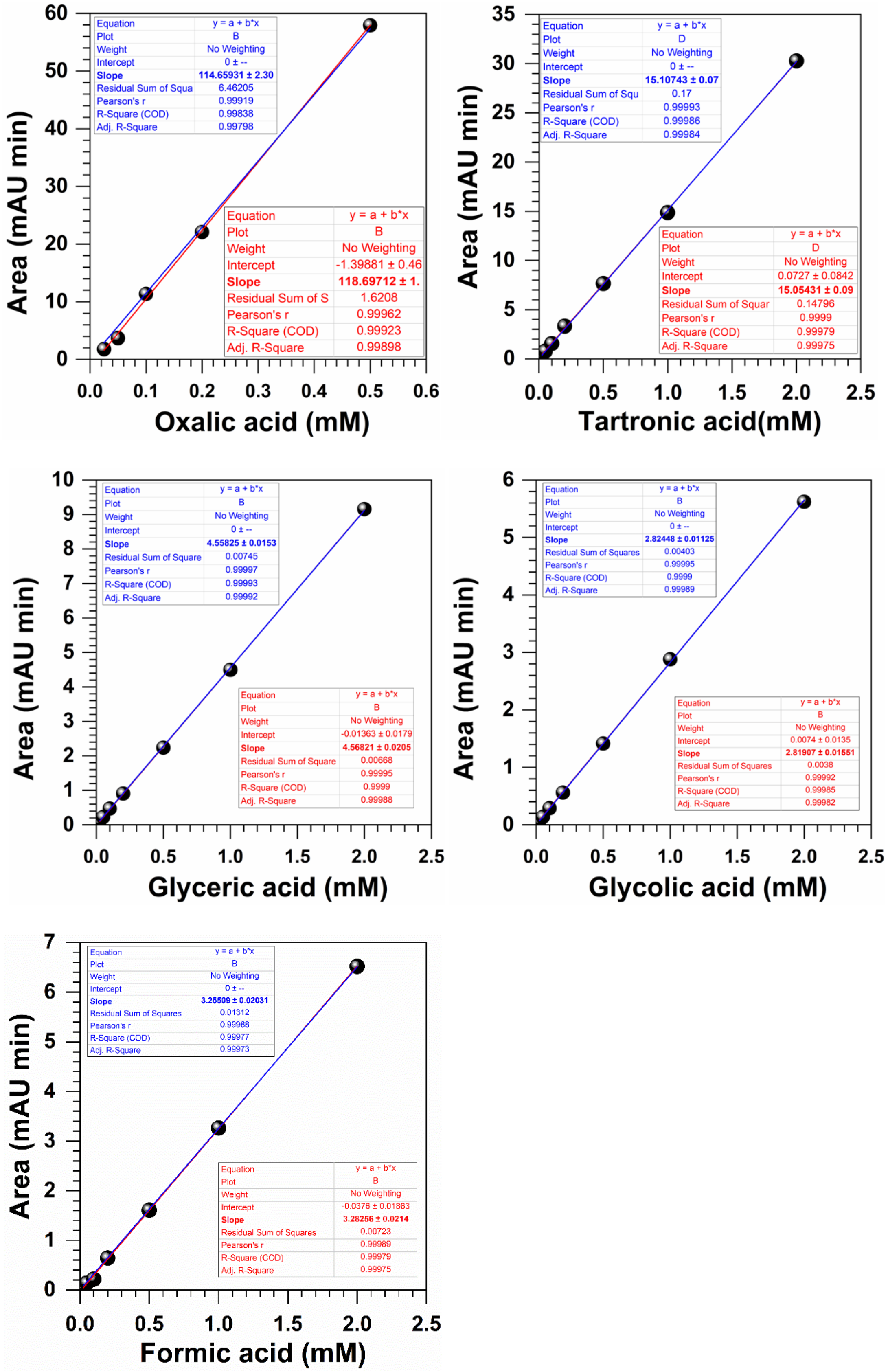

Figure S12. Calibration curves for expected standard reactants.

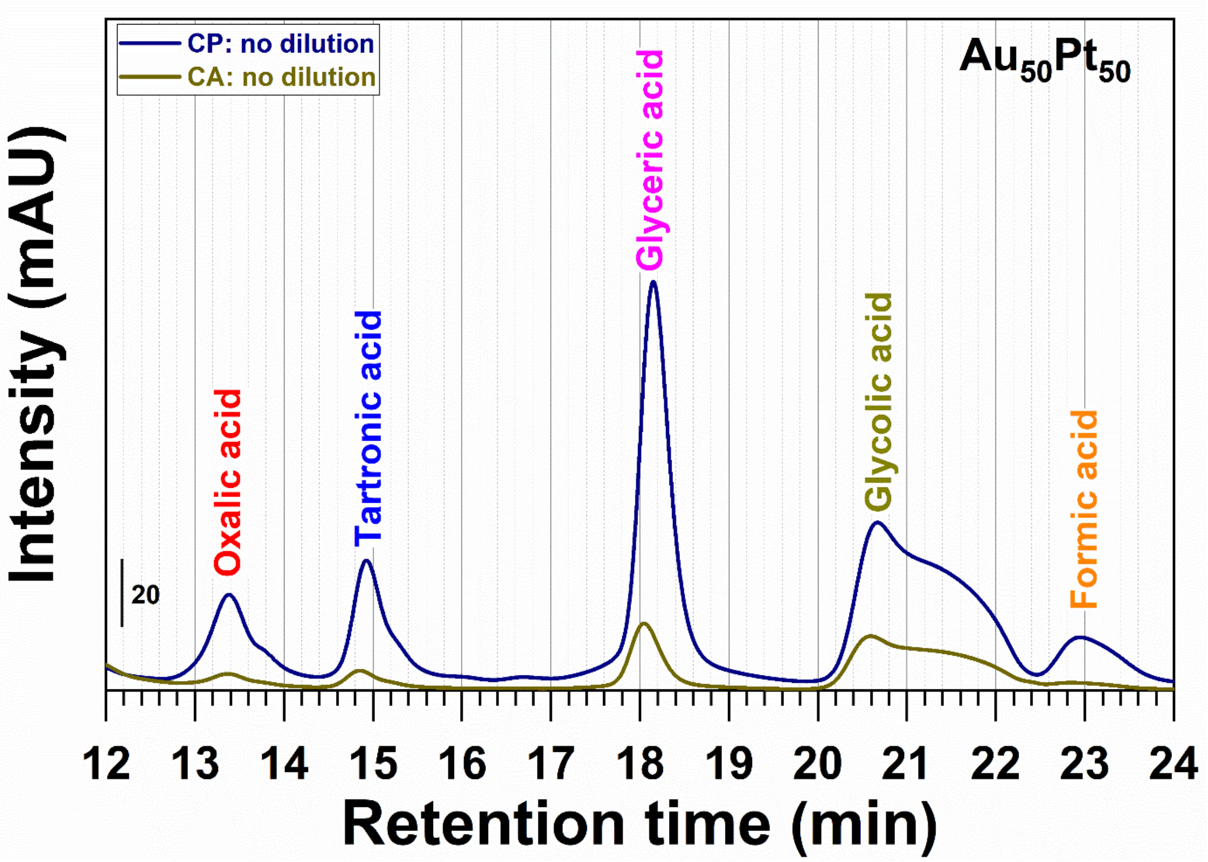

Figure. S13. Chromatogram obtained by HPLC of an electrolyte sample taken after GOR including an exemplifying product assignment. The example corresponds to a sample taken after 1 h electrolysis performed at 20 mA for CP and 0.89 V vs RHE for CA, in 1 M NaOH containing initially 100 mM glycerol.

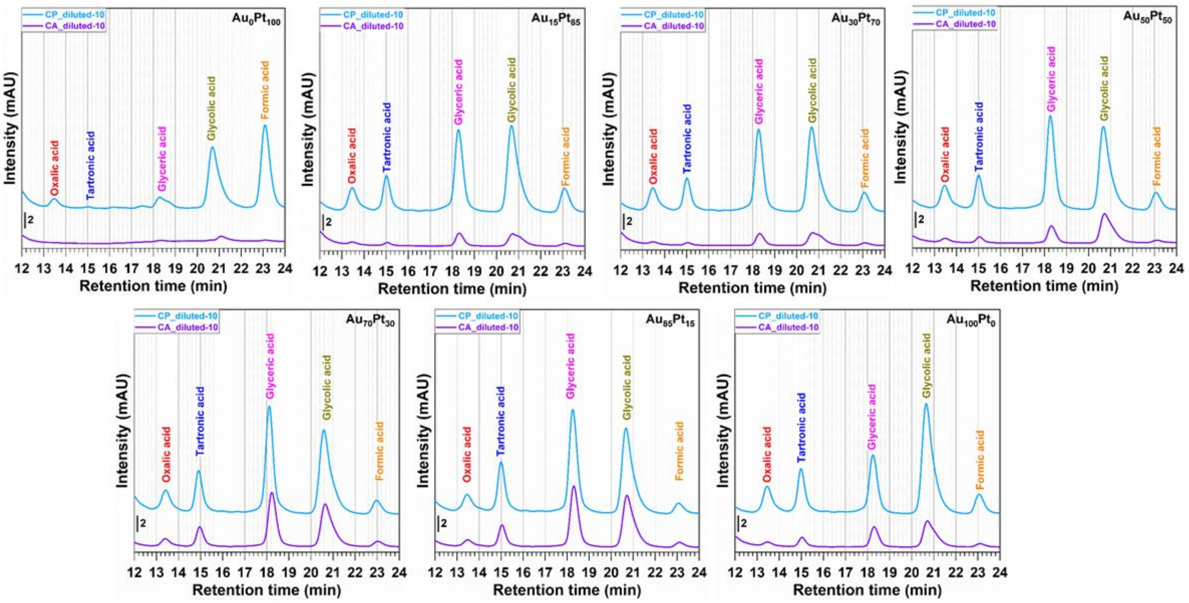

Figure. S14. Diluted HPLC chromatogram of electrolyte after glycerol oxidation reaction (GOR) in H-type cell. This figure illustrates chromatograms of samples post 1-hour electrolysis at 20 mA for CP and 0.89 V vs RHE for CA, conducted in 1 M NaOH containing 1 M glycerol initially. The chromatograms highlight the product distribution and identification.

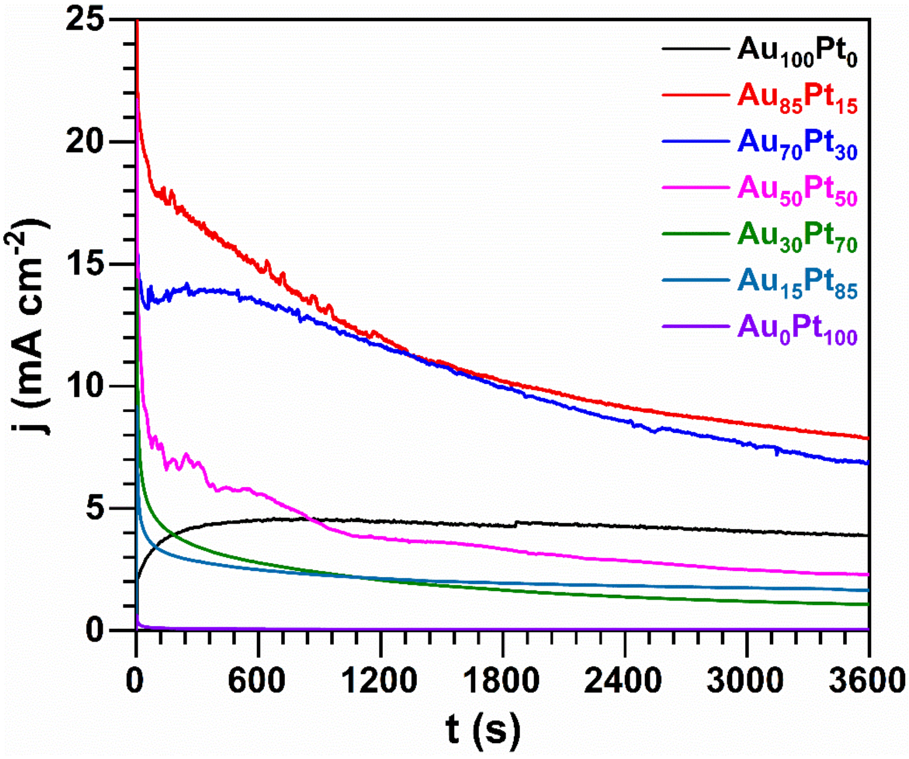

Figure. S15. CA curve during bulk electrolysis in a H-type cell at xx V vs RHE (1 M NaOH, 1 M glycerol, 25 °C, hydroxide AEM).

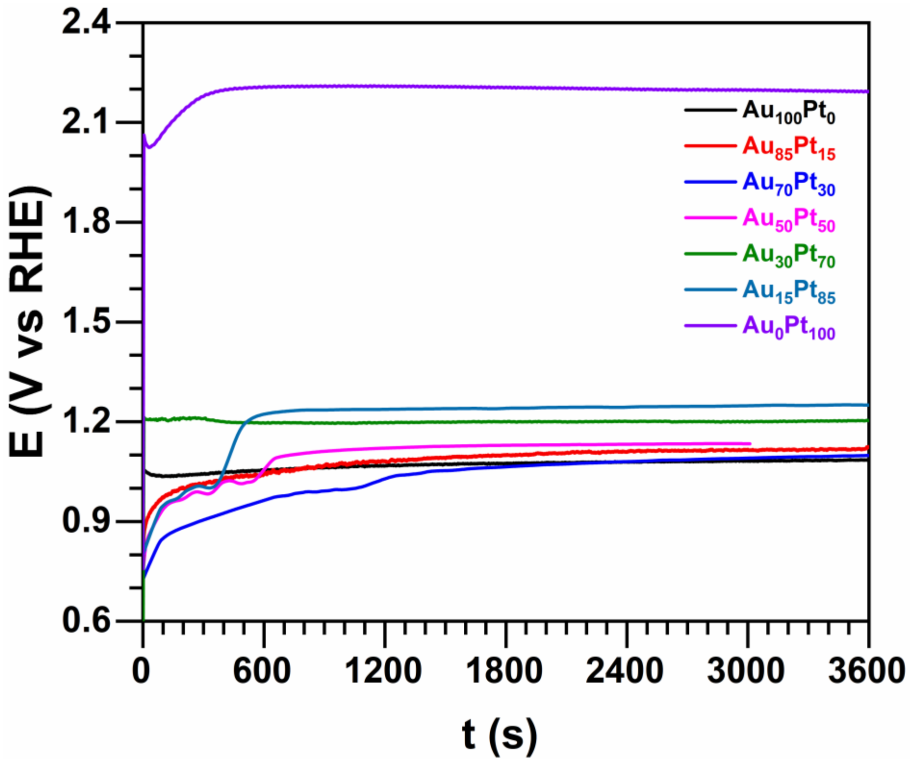

Figure. S16. CP curve during bulk electrolysis in a H-type cell at 20 mA cm^{-2} (1 M NaOH, 1 M glycerol, 25 °C, hydroxide AEM).

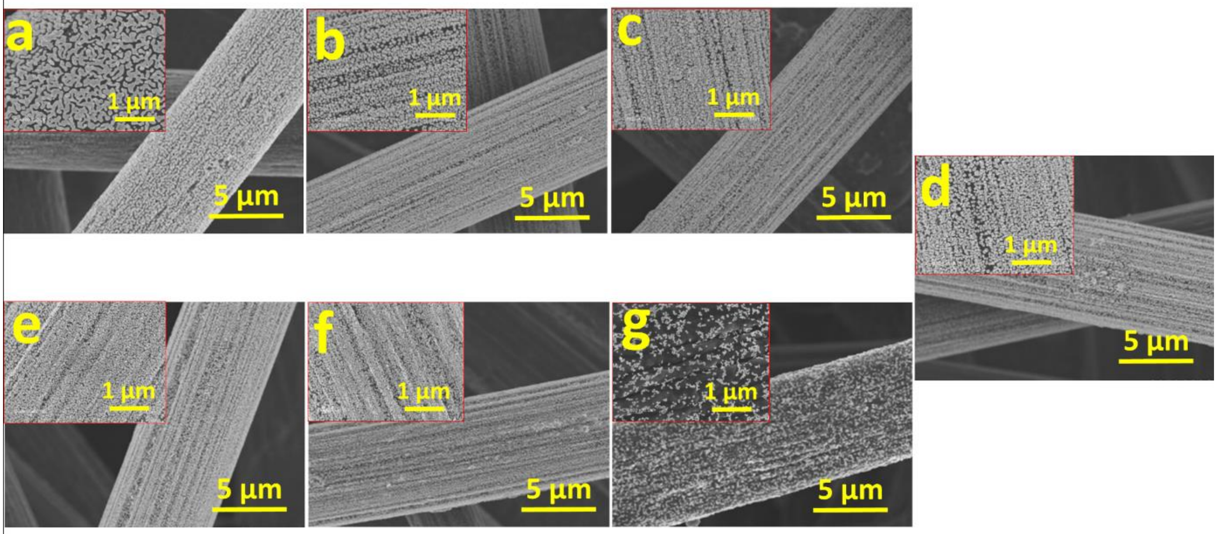

Figure S17. Post-Mortem SEM Images of GDE-Au-Pt Electrodes. This figure presents SEM images showing the morphology of the electrodes after use, with compositions as follows: (a) GDE-$Au_{100}Pt_0$, b) GDE-$Au_{85}Pt_{15}$, c) GDE-$Au_{70}Pt_{30}$, d) GDE-$Au_{50}Pt_{50}$, e) GDE-$Au_{30}Pt_{70}$, f) GDE-$Au_{15}Pt_{85}$, and g) GDE-Au_0Pt_{100}.

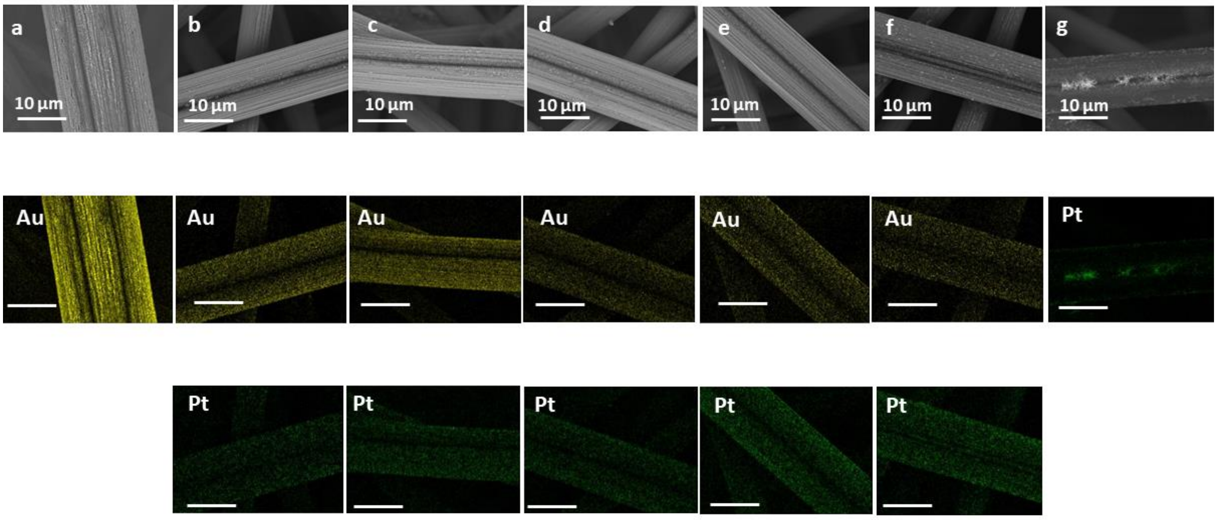

Figure S18. Post-Mortem SEM-EDX Mapping of GDE-Au-Pt Electrodes. Each panel shows the elemental distribution for different metal compositions: a) GDE-$Au_{100}Pt_{0}$, b) GDE-$Au_{85}Pt_{15}$, c) GDE-$Au_{70}Pt_{30}$, d) GDE-$Au_{30}Pt_{70}$, e) GDE-$Au_{15}Pt_{85}$, and f) GDE-Au_{0} Pt_{100}.

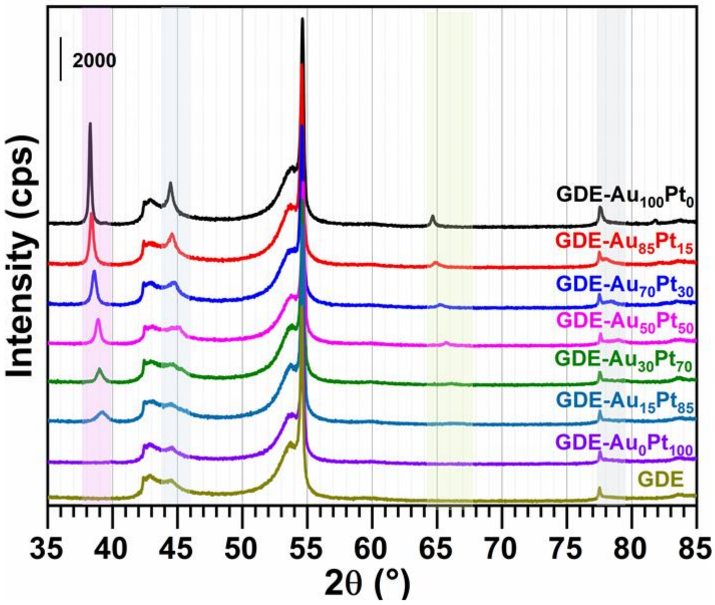

Figure S19. Post Mortem XRD Analysis of GDE-$Au_{100-x}Pt_x$ Electrodes (after 1 h glycerol electrooxidation (1 M glycerol) using CP methodology (j_{apply}= 20 mA cm^{-2}, 1 M NaOH, 50 °C). This figure illustrates the X-ray diffraction patterns of GDE-$Au_{100-x}Pt_x$ electrodes after usage, revealing their crystalline structure and compositional changes.

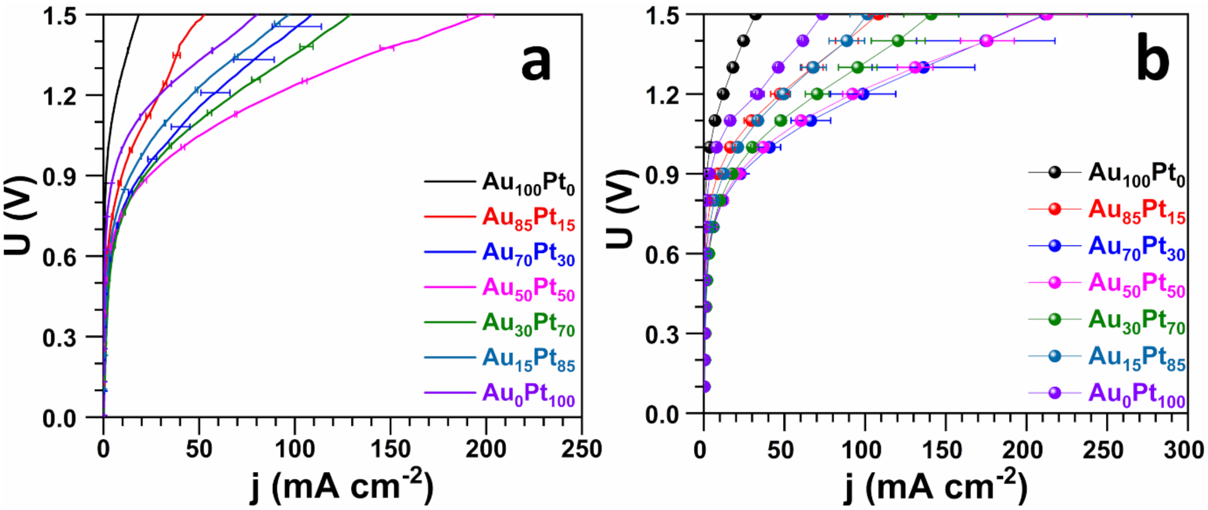

Figure S20. Polarization curves with error bars for GDE-$Au_{100-x}Pt_x$ ‖ GDE-$Au_{100-x}Pt_x$ Zero-gap glycerol electrolyzer: (a) the linear sweep voltammetry (LSV) strategy and (b) the staircase chronoamperometry strategy, highlighting performance under each method. Catholyte: 1 M NaOH (45 mL min^{-1}, 50 °C). Anolyte: 1 M NaOH + 1 M glycerol (23 mL min^{-1}, 50 °C). Hydroxide anion exchange membrane: Sustainion® X37-50 grade RT (5 cm^2).

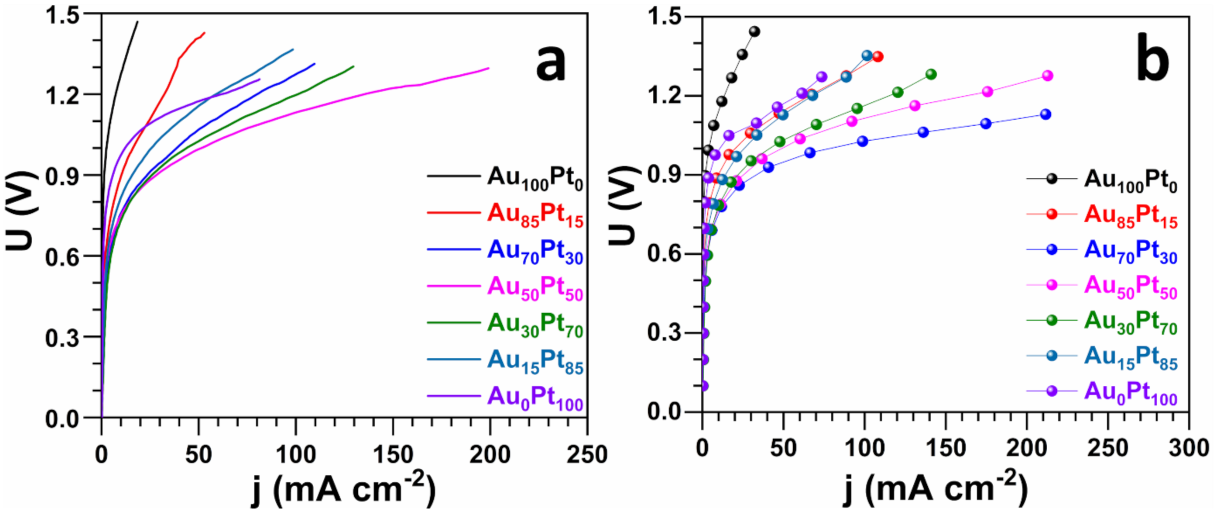

Figure S21. Polarization Curves for GDE-$Au_{100-x}Pt_x$ ‖ GDE-$Au_{100-x}Pt_x$ Zero-gap Glycerol Electrolyzer, the cell voltage were iR-drop corrected: (a) LSV and (b) the staircase chronoamperometry strategy. Catholyte: 1 M NaOH (45 mL min^{-1}, 50 °C). Anolyte: 1 M NaOH + 1 M glycerol (23 mL min^{-1}, 50 °C). Hydroxide anion exchange membrane: Sustainion® X37-50 grade RT (5 cm^2).

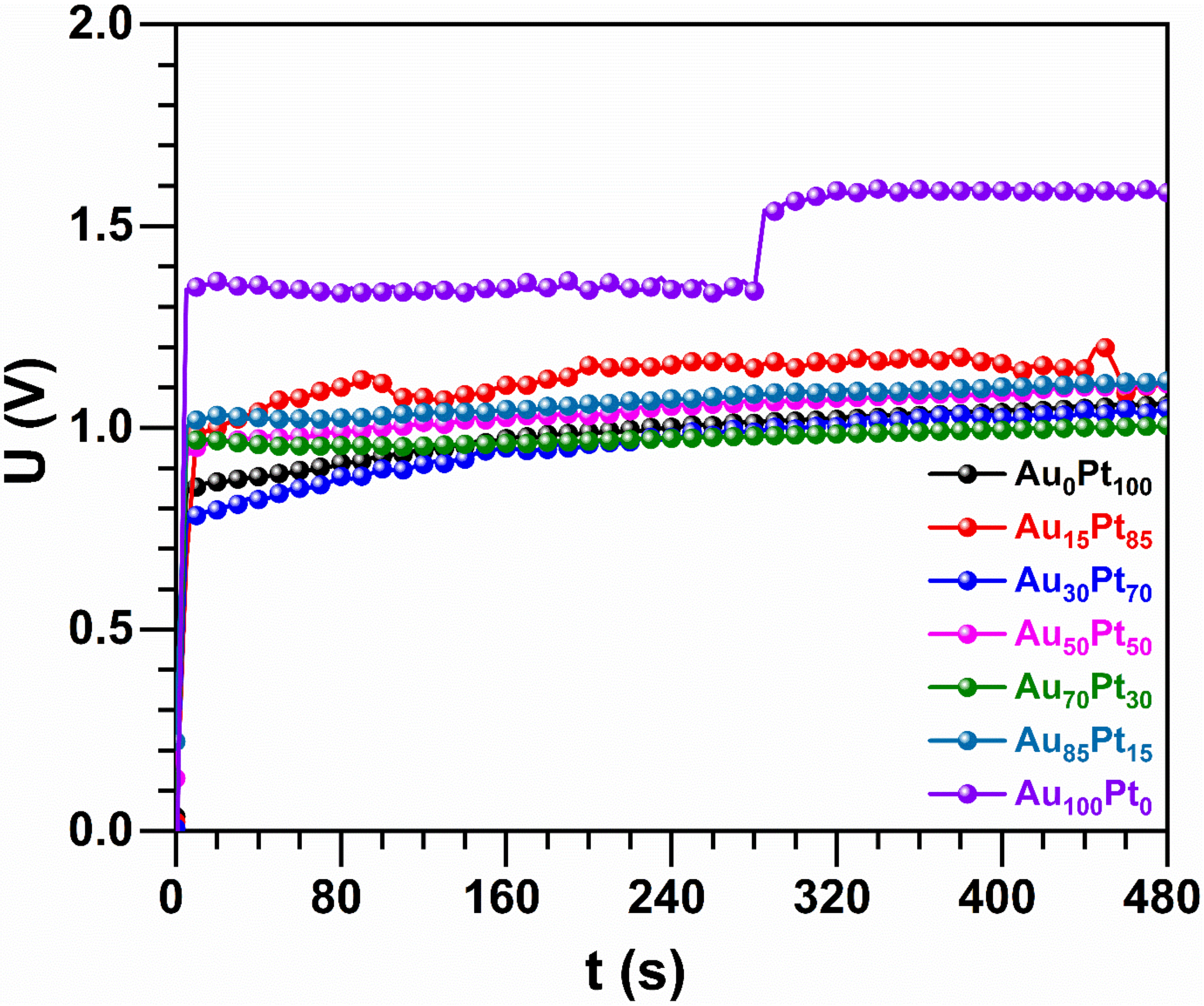

Figure S22. Evolution of cell voltage for GDE-$Au_{100-x}Pt_x$ ǁ GDE-$Au_{100-x}Pt_x$ (iR-drop uncorrected) at a current density of 20 mA cm^{-2} (0.1 A).

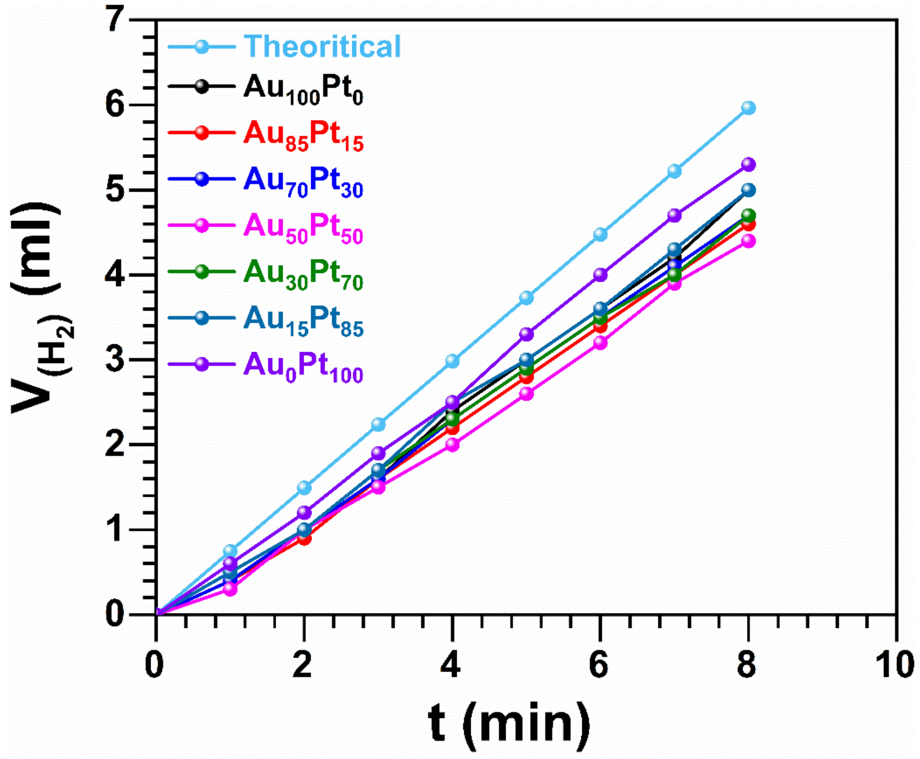

Figure S23. Comparison of hydrogen flow rate from electrolyzer cathodic outlet to theoretical production rates at a current density of 20 mA cm^{-2} (0.1 A).

References

1 C. C. Lima, M. V. F. Rodrigues, A. F. M. Neto, C. R. Zanata, C. T. G. V. M. T. Pires, L. S. Costa, J. Solla-Gullón and P. S. Fernández, *Appl. Catal. B: Env.*, 2020, **279**, 119369.

2 R. Boukil, N. Tuleushova, D. Cot, B. Rebiere, V. Bonniol, J. Cambedouzou, S. Tingry, D. Cornu and Y. Holade, *J. Mater. Chem. A*, 2020, **8**, 8848–8856.

3 H. Du, K. Wang, P. Tsiakaras and P. K. Shen, *Appl. Catal. B: Env.*, 2019, **258**, 117951.

4 Y. Zhou, Y. Shen, J. Xi and X. Luo, *ACS Appl. Mater. Interfaces*, 2019, **11**, 28953–28959.

5 Y. Zhou, Y. Shen and J. Xi, *A Appl. Catal. B: Env.*, 2019, **245**, 604–612.

6 Z. Chen, C. Liu, X. Zhao, H. Yan, J. Li, P. Lyu, Y. Du, S. Xi, K. Chi, X. Chi, H. Xu, X. Li, W. Fu, K. Leng, S. J. Pennycook, S. Wang and K. P. Loh, *Adv. Mater.s*, 2019, **31**, 1804763.

7 H. Xu, J. Wei, M. Zhang, C. Wang, Y. Shiraishi, J. Guo and Y. Du, *J. Mater. Chem. A*, 2018, **6**, 24418–24424.

8 R. G. Da Silva, S. Aquino Neto, K. B. Kokoh and A. R. De Andrade, *J. Power Sources*, 2017, **351**, 174–182.

9 H. Xu, J. Wang, B. Yan, K. Zhang, S. Li, C. Wang, Y. Shiraishi, Y. Du and P. Yang, *Nanoscale*, 2017, **9**, 12996–13003.

10 L. M. Palma, T. S. Almeida, C. Morais, T. W. Napporn, K. B. Kokoh and A. R. de Andrade, *ChemElectroChem*, 2017, **4**, 39–45.

11 J. González-Cobos, S. Baranton and C. Coutanceau, *ChemElectroChem*, 2016, **3**, 1694–1704.

12 C. Wang, X. Jiang, Q. Liu, J. Ding, J. Zhou, Y. Tang, G. Fu and J.-M. Lee, *Mater. Chem. Front.*, 2024, **8**, 265–273.

13 Y. Qiu, Z. Wen, C. Jiang, X. Wu, R. Si, J. Bao, Q. Zhang, L. Gu, J. Tang and X. Guo, *Small*, 2019, **15**, 1900014.

14 C. Zhang, B. Chen, D. Mei and X. Liang, *J. Mater. Chem. A*, 2019, **7**, 5475–5481.

15 W. Wu, Z. Tang, K. Wang, Z. Liu, L. Li and S. Chen, *Electrochim. Acta*, 2018, **260**, 168–176.

16 L. Huang, Y. Hou, Z. Yu, Z. Peng, L. Wang, J. Huang, B. Zhang, L. Qian, L. Wu and Z. Li, *Int. J. Hydrogen Energy*, 2017, **42**, 9458–9466.

17 R. Kavian, S.-I. Choi, J. Park, T. Liu, H.-C. Peng, N. Lu, J. Wang, M. J. Kim, Y. Xia and S. W. Lee, *J. Mater. Chem. A*, 2016, **4**, 12392–12397.

18 W. Luo, H. Tian, Q. Li, G. Meng, Z. Chang, C. Chen, R. Shen, X. Yu, L. Zhu, F. Kong, X. Cui and J. Shi, *Adv. Funct. Mater.*, 2024, **34**, 2306995.

19 Y. Xu, T. Liu, K. Shi, H. Yu, K. Deng, Z. Wang, X. Li, L. Wang and H. Wang, *Chem. Commun.*, 2023, **59**, 1817–1820.

20 Q. Qian, X. He, Z. Li, Y. Chen, Y. Feng, M. Cheng, H. Zhang, W. Wang, C. Xiao, G. Zhang and Y. Xie, *Adv. Mater.*, 2023, **35**, 2300935.

21 Z. He, J. Hwang, Z. Gong, M. Zhou, N. Zhang, X. Kang, J. W. Han and Y. Chen, *Nat Commun*, 2022, **13**, 3777.

22 L. Fan, Y. Ji, G. Wang, J. Chen, K. Chen, X. Liu and Z. Wen, *J. Am. Chem. Soc.*, 2022, **144**, 7224–7235.

23 X. Yu, E. C. Dos Santos, J. White, G. Salazar-Alvarez, L. G. M. Pettersson, A. Cornell and M. Johnsson, *Small*, 2021, **17**, 2104288.

24 Y. Li, X. Wei, S. Han, L. Chen and J. Shi, *Angew. Chem., Int. Ed.*, 2021, **133**, 21634–21642.

25 Y. Li, X. Wei, L. Chen, J. Shi and M. He, *Nat. Commun.*, 2019, **10**, 5335.

26 A. Cassani, N. Tuleushova, Q. Wang, H. Guesmi, V. Bonniol, J. Cambedouzou, S. Tingry, M. Bechelany, D. Cornu, Y. Holade, *ACS Appl. Energy Mater.* **2021**, *4*, 9944-9960.
